# Elucidating colorectal cancer-associated bacteria through profiling of minimally perturbed tissue-associated microbiota

**DOI:** 10.3389/fcimb.2023.1216024

**Published:** 2023-08-01

**Authors:** Hironori Fukuoka, Dieter M. Tourlousse, Akiko Ohashi, Shinsuke Suzuki, Kazuya Nakagawa, Mayumi Ozawa, Atsushi Ishibe, Itaru Endo, Yuji Sekiguchi

**Affiliations:** ^1^ Department of Gastroenterological Surgery, Yokohama City University Hospital, Yokohama, Japan; ^2^ Biomedical Research Institute, National Institute of Advanced Industrial Science and Technology (AIST), Tsukuba, Japan; ^3^ Department of Surgery, Fujisawa Shonandai Hospital, Fujisawa, Japan

**Keywords:** colorectal cancer, mucosal microbiota, 16S rRNA gene amplicon sequencing, metagenome-assembled genomes, bowel preparation, unprepped colon resection

## Abstract

Sequencing-based interrogation of gut microbiota is a valuable approach for detecting microbes associated with colorectal cancer (CRC); however, such studies are often confounded by the effect of bowel preparation. In this study, we evaluated the viability of identifying CRC-associated mucosal bacteria through centimeter-scale profiling of the microbiota in tumors and adjacent noncancerous tissue from eleven patients who underwent colonic resection without preoperative bowel preparation. High-throughput 16S rRNA gene sequencing revealed that differences between on- and off-tumor microbiota varied considerably among patients. For some patients, phylotypes affiliated with genera previously implicated in colorectal carcinogenesis, as well as genera with less well-understood roles in CRC, were enriched in tumor tissue, whereas for other patients, on- and off-tumor microbiota were very similar. Notably, the enrichment of phylotypes in tumor-associated mucosa was highly localized and no longer apparent even a few centimeters away from the tumor. Through short-term liquid culturing and metagenomics, we further generated more than one-hundred metagenome-assembled genomes, several representing bacteria that were enriched in on-tumor samples. This is one of the first studies to analyze largely unperturbed mucosal microbiota in tissue samples from the resected colons of unprepped CRC patients. Future studies with larger cohorts are expected to clarify the causes and consequences of the observed variability in the emergence of tumor-localized microbiota among patients.

## 1 Introduction

Colorectal cancer (CRC) is one of the most common cancers worldwide. In Japan, the number of annual deaths due to CRC has increased steadily and exceeded 50,000 in 2016 (Hashiguchi et al., 2020). Colon cancer has a multifactorial etiology, and common risk factors include genetic mutations, unhealthy diet and lifestyle, and disruption of inflammatory processes (O’Keefe, 2016). Furthermore, a range of bacteria, including *Fusobacterium nucleatum* (Rubinstein et al., 2013), *Streptococcus gallolyticus* (Pasquereau-Kotula et al., 2018), enterotoxigenic *Bacteroides fragilis* (Boleij et al., 2015), and *Peptostreptococcus anaerobius* (Long et al., 2019), have been implicated in colonic tumorigenesis, and the mechanisms underlying their oncogenic effects are starting to be understood.

High-throughput sequencing studies have substantially improved our understanding of the association between CRC and gut microbiota but a unified picture has not yet emerged (Helmink et al., 2019). This may partly be due to the different types of samples and protocols adopted across studies (Rezasoltani et al., 2019). Fecal samples are commonly used because of their ease of collection but may be less informative for identifying disease-specific microbiota perturbations than colonic tissue samples (Gorkiewicz et al., 2013; Ringel et al., 2015). Current studies on tissue-associated microbiota in patients with CRC typically involve bowel preparation (cleansing) before sampling (Loke et al., 2018; Murphy et al., 2021). However, bowel preparation can alter intestinal microbiota (Mai et al., 2006; Harrell et al., 2012; Jalanka et al., 2015), and short-term changes in microbiome diversity and composition due to bowel preparation have been shown to introduce confounding effects in gastrointestinal microbiota studies (Shobar et al., 2016). The type of bowel cleansing procedures may also vary depending on the location of the tumor and healthcare facility, further complicating the interpretation of observations from different cohorts. More studies are thus needed that analyze microbiota in colonic tissue of CRC patients who did not undergo bowel preparation prior to tissue sample collection (that is, unprepped patients).

In this pilot study, we evaluated the feasibility of identifying CRC-associated bacteria by profiling mucosal microbiota in tumors and adjacent noncancerous colonic tissues from eleven patients who underwent colonic resection without preoperative bowel preparation. Specifically, we (i) compared on- and off-tumor mucosal microbiota, including evaluation of the centimeter-scale spatial distribution of taxa around the tumor for some patients and (ii) generated genome assemblies (that is, metagenome-assembled genomes, MAGs) of major tumor-associated bacteria through short-term liquid culturing and metagenome sequencing.

## 2 Materials and methods

### 2.1 Patient cohort and tissue sample collection

Patients diagnosed with CRC and scheduled for colonic resection at Yokohama City University Hospital (Yokohama, Kanagawa, Japan) were recruited. Between October 2019 and March 2021, colorectal surgery was performed on 155 patients, with 11 patients undergoing surgery without preoperative bowel cleansing, in accordance with the Enhanced Recovery After Surgery (ERAS^®^) protocols (Gustafsson et al., 2019); samples from these patients were analyzed in this study. The patients were only administered intravenous antibiotics (cefmetazole sodium) approximately 30 min before surgery, except for patient D (**Supplementary Table S1**). Tissue samples were collected from the resected colons within 30 to 50 min of surgery. The specimens were handled on a clean surface using sterile tools, including scissors, biopsy forceps, gauze, and surgical knives. The resected intestinal tracts were opened longitudinally using scissors, taking care not to disrupt the cancerous tissue and to prevent the mixing of intraluminal fecal material. Rinsing of the tissues was omitted in order not to disturb the microbiota, and mucosal tissue of the tumor and surrounding areas with normal appearance were resected using scissors after careful removal of solid fecal material from the sampling areas. The collected tissue samples had an area of approximately 10 to 20 mm^2^ and a depth of several millimeters (including the mucosal and submucosal layers). The samples were placed in cryogenic tubes, snap-frozen in liquid nitrogen, and frozen until further analysis.

### 2.2 Ethics approval and informed consent

The study protocol was approved by the Ethics Committee of Yokohama City University (B190600051, F220600030) and was registered in the University Hospital Medical Information Network (UMIN) under UMIN000038703, and in the Japan Registry of Clinical Trials (jRCT) under jRCT1030220239. All participants provided written informed consent to participate in this study and publish their clinical data.

### 2.3 Enrichment cultures

Enrichment cultures were set up in 50-mL serum bottles under an atmosphere of N_2_/CO_2_ (80:20, v/v). The culture medium consisted of the basal medium described by Sekiguchi et al. (2000), supplemented with autoclaved yeast extract (0.5%, w/v, final concentration), peptone (0.5%), brain-heart infusion (1%), and ascorbic acid (0.1%). For inoculation, tissue samples were broken up with a 25-gauge syringe needle in 1 mL of basal medium, vortexed, and 400 μL was added as an inoculant to 20 mL of medium. Cultures were incubated anaerobically for approximately 24 h at 37°C, under static conditions in the dark.

The above-described medium was selected based on a comparison with YCFA medium (medium 1130; https://www.jcm.riken.jp/cgi-bin/jcm/jcm_grmd?GRMD=1130). The latter yielded considerably less microbial biomass, and was thus less effective in reducing human DNA content, with an average of roughly 75% of human reads remaining in the resultant metagenomic sequencing data across cultures. Furthermore, we initially evaluated the depletion of methylated human DNA using a commercial kit (NEBNext Microbiome DNA Enrichment Kit) but this was not effective for our samples, as human DNA still accounted for more than 95% of sequencing reads (data not shown).

### 2.4 Extraction of DNA from colonic tissue and enrichment cultures

Tissue samples were crushed using a CP02 cryoPREP Automated Dry Pulverizer (Covaris). To this end, the samples were placed in the center of a tissueTUBE (TT 1; Covaris), flash-frozen with liquid nitrogen, and pulverized by a single action of the cryoPREP hammer at an impact level of 6. Samples were then transferred to Eppendorf tubes and resuspended in 300 μL of phosphate-buffered saline (PBS). DNA was extracted using a phenol-chloroform protocol described previously (Tourlousse et al., 2021). In short, biomass pellets were resuspended in 300 μL of 100 mM NaH_2_PO_4_ (pH 8.0), 300 μL of lysis buffer (100 mM of NaCl, 500 mM of Tris-HCl, 10% of sodium dodecyl sulfate, pH 8.0) and 300 μL of phenol-chloroform-isoamyl alcohol (PCI, 25:24:1, v:v:v), followed by addition of 1.2 g of 0.1-mm autoclaved Zirconia beads. Cell lysis was performed by bead-beating using a FastPrep-24 instrument (MP Biomedicals) for 60 s at a speed of 6 m/s. Following incubation for 10 min at 60°C, samples were centrifuged for 5 min at 14,000 × *g* and 600 μL of supernatant was recovered. RNA was digested with 0.01 volumes of RNase A (10 mg/ml) for 10 min at 37°C. An equal volume of PCI was then added, the samples were mixed by vortexing, and the aqueous phase with DNA recovered after centrifugation (20,000 × *g*; 23°C; 5 min); this step was repeated twice. Subsequently, an equal volume of chloroform-isoamyl alcohol (24:1, v/v) was added, the samples were vortexed, and the aqueous phase was recovered after centrifugation (20,000 × *g*; 23°C; 5 min). DNA was precipitated by the addition of 0.1 volumes of 3 M sodium acetate (pH 5.2) and an equal volume of isopropanol, followed by centrifugation at 20,000 × *g* for 30 min at 4°C. Recovered DNA pellets were washed with 70% ethanol, air-dried, and dissolved in elution buffer (Qiagen EB solution; 10 mM Tris-HCl, pH 8.5). For the enrichment cultures, DNA was extracted using the ISOSPIN Fecal DNA kit (NipponGene) following the manufacturer’s instructions, as detailed previously (Tourlousse et al., 2021).

### 2.5 16S rRNA gene amplicon and metagenome sequencing

Amplicon libraries of the V4 hypervariable region of the 16S rRNA gene were generated using Illumina’s two-step tailed PCR protocol. First-round PCRs (20 μL) contained 5 units of AmpliTaq Gold DNA Polymerase LD, 1× Gold Buffer, 1.5 mM of MgCl_2_, 200 μM of each deoxynucleotide (dNTP), 500 nM each of forward primer (5′-TCGTCGGCAGCGTCAGATGTGTATAAGAGACAGGTGYCAGCMGCCGCGGTAA-3′, the locus-specific 515F primer region is underlined) and reverse primer (GTCTCGTGGGCTCGGAGATGTGTATAAGAGACAGGGACTACNVGGGTWTCTAAT; the 806R primer region is underlined), and 2 μL of DNA template. Thermal cycling conditions were as follows: 95°C for 10 min; 25 cycles at 95°C for 45 s, 50°C for 45 s, and 72°C for 1 min; and 72°C for 5 min. The amplicons were cleaned up using the Agencourt AMPure XP PCR Purification system, following the manufacturer’s instructions with a 1-to-1 bead-to-sample ratio, and eluted with 10 mM Tris-HCl (pH 8.5). The Nextera XT Index Kit was used to attach dual indexes and sequencing adapters in PCRs (50 μL) containing 1× KAPA HiFi HotStart ReadyMix, 5 μL each of Index 1 and 2 primers, and 5 μL of purified first-round PCR products. Thermal cycling conditions were as follows: 95°C for 3 min; 8 cycles at 95°C for 30 s, 55°C for 30 s, and 72°C for 30 s; and 72°C for 5 min. The amplicons were purified as described above and quantified using the D1000 ScreenTape Assay system and a 2200 TapeStation instrument (Agilent). Libraries were pooled at equimolar concentrations, supplemented with phiX control DNA (~30%), and sequenced on a MiSeq instrument using V2 chemistry (2×251 bp paired-end reads).

Libraries for shotgun metagenomics were prepared using the ThruPLEX DNA-Seq Kit (Takara Bio) as previously described (Tourlousse et al., 2021). Sequencing was performed on a NextSeq 500 instrument using a NextSeq 500/550 Mid Output Kit v2.5 (2×151 bp paired-end reads). Binary base call (BCL) files were converted to FASTQ format, with concurrent library demultiplexing, using Illumina’s bcl2fastq Conversion Software v2.20.0.422, with default settings.

### 2.6 Amplicon sequence data processing and analysis

Primer sequences were trimmed using Cutadapt v3.5 (Martin, 2011), with flags -u 3 -U 4 -g ^YCAGCMGCCGCGGTRA -G ^TACNVGGGTWTCTAAK -error-rate 0.2 -no-indels -discard-untrimmed -max-n 0 -minimum -length 225. Reads were then truncated and filtered based on expected errors using the DADA2’s v1.22 (Callahan et al., 2016) filterAndTrim function, with parameters truncLen = c(160,180), maxN = c(0,0), maxEE = c(4,4), truncQ = c(2,2), rm.phix = TRUE. Subsequent steps, namely the learning of error models (function learnErrors), denoising (function dada), and merging (function mergePairs) of forward and reverse reads, were performed for data from individual sequencing runs separately, with default settings. Resulting sequence tables were then combined (function mergeSequenceTables), and bimeras were removed using function removeBimeraDenovo, with method = “consensus.” Taxonomy was assigned against the SILVA database (Yilmaz et al., 2014) (file “silva_nr99_v138.1_train_set.fa.gz” obtained from 10.5281/zenodo.4587955) using function assignTaxonomy, with a default minimum bootstrap confidence of 50. The final ASV table was generated by culling ASVs that had an aberrant length (< 245 bp or > 260 bp), lacked taxonomic assignment at the kingdom or phylum level, or were classified as mitochondria. Comparison of ASVs against the LTP database (file “LTP_01_2022_compressed.fasta” obtained from https://imedea.uib-csic.es/mmg/ltp/#Downloads) was performed using Blastn (v2.13.0+), with flags -max_target_seqs 100 -perc_identity 80 -qcov_hsp_perc 95; alignment(s) with the highest percent sequence identity were retained for each ASV.

### 2.7 Metagenome sequence data processing and analysis

Metagenome sequencing reads were preprocessed using fastp v0.20.0 (Chen et al., 2018), with flags -trim_front1 5 -trim_front2 5 -trim_tail1 1 -trim_tail2 1 -cut_right -cut_right_window_size 4 -cut_right_mean_quality 15 -trim_poly_x -poly_x_min_len 10 -n_base_limit 0 -low_complexity_filter -length_required 75. Reads derived from human DNA were identified and removed using BMTagger v3.101 (ftp://ftp.ncbi.nlm.nih.gov/pub/agarwala/bmtagger/), using human genome assembly GRCh38 as reference. The retained reads were merged using the BBMap’s v38.82 (https://sourceforge.net/projects/bbmap/) bbmerge.sh script with default parameters.

Assembly was performed with MEGAHIT v1.2.9 (Li et al., 2015) with default parameters, using the merged and unmerged paired reads as inputs. For metagenome binning, paired reads were mapped against the assembled contigs using bowtie2 v2.4.3 (Langmead and Salzberg, 2012), with default settings, and resultant alignment files were processed using samtools v1.14 (Li et al., 2009). Binning was performed using MetaBAT 2’s v2.15 (Kang et al., 2019) runMetaBat.sh script, with -m 1500.

The quality of recovered MAGs was evaluated using lineage-specific single-copy marker gene sets with CheckM’s v1.1.3 (Parks et al., 2015) lineage_wf command. Taxonomic classification was performed against the Genome Taxonomy Database release 207 using the GTDB-Tk v2.0.0 (Chaumeil et al., 2019) with option -full_tree. Genome-based phylogenetic analysis was performed using the GTDB-Tk’s de_novo_wf command.

Genomes were annotated using DFAST v1.2.15 (Tanizawa et al., 2018) with default parameters. For MAGs classified to the genus *Fusobacterium* within the GTDB (see above), putative fadA proteins encoded in the genomes were identified by searching DFAST predicted protein sequences against the Pfam database (Mistry et al., 2021) release 35 using HMMER’s v3.3.2 (Eddy, 2011) hmmsearch command. Protein sequences assigned to the protein family PF09403 were compared against NCBI’s non-redundant protein sequences with protein Blast (blastp; performed on August 21, 2022).

### 2.8 Linking of amplicon sequence variants and metagenome-assembled genomes

ASVs and MAGs were linked using MarkerMAG v1.1.18 (Song et al., 2022). More specifically, 16S rRNA gene sequences were reconstructed from quality-controlled Illumina short reads using MarkerMAG’s matam_16s command, specifying options -pct 1,5,10,25,50,75,100 -i 0.999. Two related reference databases were used: the SILVA database (release 138, file SILVA_138.1_SSURef_NR99_tax_silva.fasta) and SortMeRNA’s v4.3 (Kopylova et al., 2012) smr_v4.3_sensitive_db_SSURef_NR99.fasta. Databases were processed using MarkerMAG’s matam_db_preprocessing.py script, with clustering_id_threshold 0.99. Reconstructed 16S rRNA genes were then linked to MAGs using MarkerMAG’s link function, with command line flags -skip_cn -no_cluster, using the assembled 16S rRNA gene sequences (clustered at 99.9% sequence identity) as markers. Reconstructed 16S rRNA gene sequences, trimmed to the V4 region by *in silico* PCR using Cutadapt, were compared against the ASV sequences using VSEARCH’s v2.18.0 (Rognes et al., 2016) usearch_global command (-maxaccepts 0 -maxrejects 0 -leftjust -rightjust).

### 2.9 Data analysis

All data were analyzed in R (R Core Team, 2022) using the packages available as part of ‘tidyverse’ (Wickham et al., 2019), including dplyr and ggplot2, for data manipulation and visualization, respectively. ASV tables were randomly subsampled (rarefied) to even depth (25,000 sequences per sample, without replacement) using vegan’s (Oksanen et al., 2020) *rrarefy* function. Beta diversity (Bray–Curtis dissimilarity) was calculated using vegan’s *vegdist* function. Principal coordinate analysis was performed with ape’s (Paradis and Schliep, 2019) *pcoa* function, based on the Bray-Curtis dissimilarity matrix. Hierarchical clustering based on square root transformed Bray-Curtis dissimilarity matrix was performed using R stats’ *hclust* function, with method = ‘ward.D2’. Permutational multivariate analysis of variance (PERMANOVA) was performed based on Bray-Curtis dissimilarities using vegan’s *adonis2* function. Alpha diversity was calculated as ASV level richness and Shannon diversity using the rarefied ASV count table as input, with vegan’s *diversity* and *specnumber* functions. To identify ASVs with significantly different abundances in on-tumor samples compared to off-tumor samples, we used general linear modeling, as implemented in the R package MaAsLin2 v1.8.0 (Mallick et al., 2021), using the rarefied ASV count table and considering tumor location (encoding as a binary categorical variable, on- and off-tumor) as fixed effects and, if applicable, patient as a random effect. Default parameter settings were used, including abundance/prevalence filtering of ASVs (min_abundance = 0.0, min_prevalence = 0.1), normalization by total-sum-scaling (normalization = “TSS”), and logarithmic transformation (transform = “LOG”). Differences were considered significant at a q-value of 0.1, i.e., the P-value after multiple testing corrections using MaAsLin2’s default Benjamini-Hochberg method.

## 3 Results

Eleven patients who underwent colonic resection without preoperative bowel preparation were analyzed in this study, namely eight male and three female patients with an average age of 71.1 years (**Table 1**). Tumors were located in the distal (from the descending colon to rectum, left-sided) and proximal colon (from the cecum to the transverse colon, right-sided) in 5 and 6 patients, respectively. Most patients (*n* = 7) were diagnosed with stage III CRC, and the remaining patients had stage II (*n* = 3) or stage I (*n* = 1) cancer. Additional patient characteristics, such as medical comorbidities and operative procedures, are provided in **Supplementary Table S1**.

**Table 1:** Characteristics of patients included in this study.

Patient	A	B	C	D	E	F	G	H	I	J	K
Gender	female	male	male	male	male	female	female	male	male	male	male
Age	73	77	68	75	69	52	70	77	79	71	71
BMI ^a^	23.2	20.7	19.2	23.8	28.0	22.5	19.6	24.4	25.3	22.0	22.4
Cancer stage ^b^	IIA	I	IIB	IIIB	IIA	IIIB	IIIC	IIIB	IIIC	IIIB	IIIB
Tumor location	right, ascending colon	right, cecum	left, sigmoid colon	left, sigmoid colon	left, sigmoid colon	left, sigmoid colon	right, ascending colon	right, transverse colon	right, ascending colon	left, rectum	right, ascending colon
Time between resection and sampling collection (min)	21–25	24–30	35–50	NA ^c^	16–25	17–25	18–27	NA ^b^	16–25	29–34	25–34
No. of samples, total (on-tumor)	28 (4)	22 (2)	9 (2)	6 (2)	6 (2)	6 (2)	6 (2)	4 (2)	4 (2)	4 (2)	3 (1)

a BMI, body mass index (in kg m^-2^).

b Cancer stage as determined according to the Union for International Cancer Control (UICC) TNM Classification of Malignant Tumors.

c NA, not available.

We obtained multiple tumor and adjacent non-tumor tissue samples from each patient, both toward the oral and anal side of the colon and at varying distances, between 1 and 10 cm, from the tumor (**Supplementary Figure S1**). Across patients, a total of 98 tissue samples were obtained, and associated microbiota compositions were measured by sequencing the V4 hypervariable region of the 16S rRNA gene. Amplicon sequence variants (ASVs) were reconstructed using DADA2 (see **Supplementary Table S2** for summary statistics) and taxonomically assigned against the SILVA database.

Colonic mucosal microbiota across patients were dominated by the phyla *Firmicutes*, *Bacteroidota*, *Proteobacteria*, and *Actinobacteriota* (**Supplementary Figure S2**), common members of the human gut microbiome. The abundance of the phylum *Fusobacteriota* varied considerably across patients, ranging from, on average, less than 1% in noncancerous tissue samples for patients I, D, A, and F to more than 10% for patients B, C, and E.

**Figure 1:**
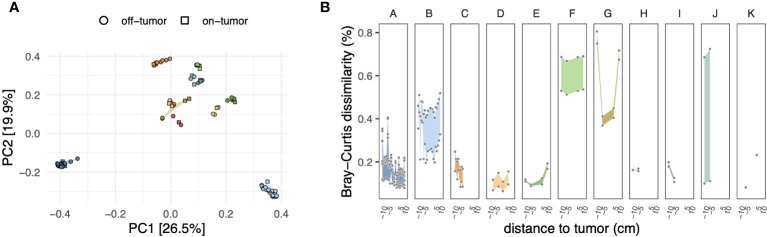
**(A)** Principal coordinate analysis (PCoA) of microbiota compositions based on the Bray–Curtis dissimilarity matrix calculated from ASV-level abundances. Symbol shapes and fill colors indicate sample locations (i.e., on- and off-tumor) and patients, respectively, as in panel **(B)** Differences in compositions of on- and off-tumor mucosal microbiota. For each patient, symbols show ASV level Bray–Curtis dissimilarities of all possible pairwise comparisons between on- and off-tumor samples, resulting in multiple data points for a given distance to the tumor depending on the number of available samples (see **Supplementary Figure S1**). Negative and positive distances to the tumor indicate tissue samples toward the oral and anal side of the colon concerning the tumor, respectively. Colored ribbons show the range of the data.

Ordination and hierarchical clustering based on ASV level Bray-Curtis dissimilarities showed that microbiota profiles clustered predominantly by the patient (**Figure 1A** and **Supplementary Figure S3**), with PERMANOVA indicating that patient-wise grouping accounted for most of the variation across microbiota profiles (R^2 = ^0.89; *P* < 0.001). Alpha diversity (ASV level richness and Shannon diversity) varied between patients but was not significantly associated with sampling location (that is, on-/off-tumor) (**Supplementary Figure S4**).

We next analyzed subject-matched (that is, paired) on- and off-tumor microbiota profiles to assess whether tumors harbored distinct microbiota compared to adjacent non-tumor tissues. At the community level, the dissimilarity between on- and off-tumor microbiota was highly variable across patients. As shown in **Figure 1B**, patients B, F, and G harbored on-tumor microbiota that was more distinct from off-tumor microbiota than the other patients. Visualization of the ASV-wise differential abundances between on- and off-tumor samples revealed a similar trend (**Supplementary Figure S5**).

**Figure 2:**
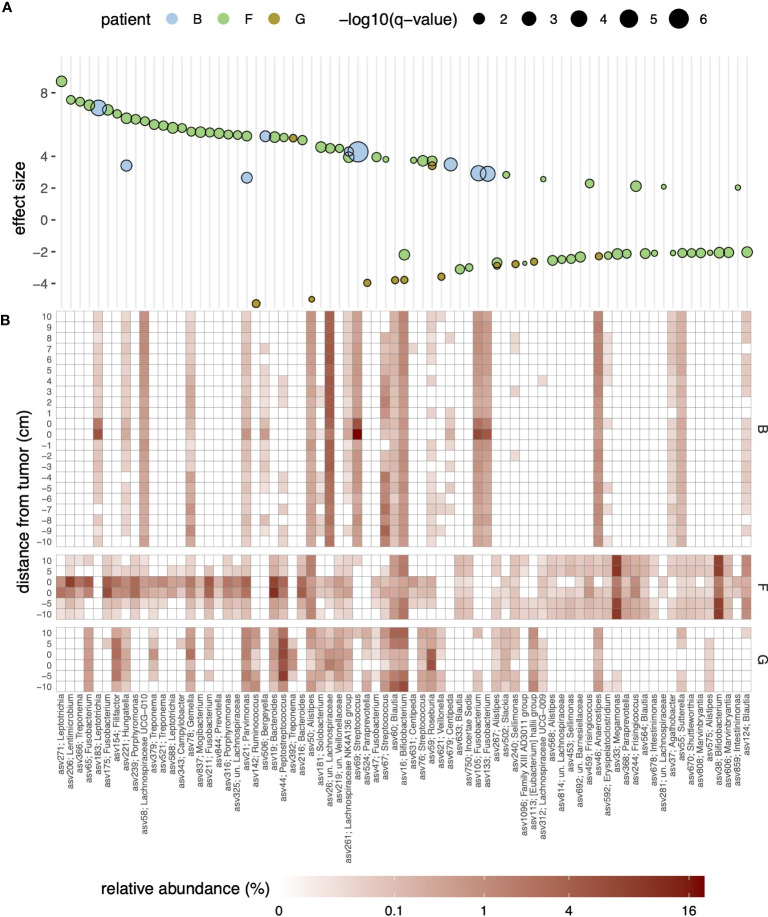
**(A)** Bubble plot of effect sizes for ASVs with significantly differential abundance in the on- and off-tumor samples for patients (B, F, G) Effect sizes were estimated using MaAsLin2 and represent log_2_-transformed fold-differences in abundances; positive and negative effect sizes indicate enrichment or depletion in the on-tumor samples, respectively. Only significant ASVs (q-value of <0.1) and an effect size (absolute value) of >2, corresponding to a four-fold difference in abundance, are shown. Symbol fill colors and sizes indicate patients and statistical significance levels (q-values), respectively. Genus-level taxonomic assignments of the ASVs against the SILVA database are indicated in the *x*-axis labels. **(B)** Heatmap of the relative abundance of the ASVs shown in panel **(A)**; fill colors reflect relative abundances in percentages.

Based on the above observations, we focused on patients with the largest dissimilarity between on- and off-tumor samples (that is, patients B, F, and G) and statistically assessed ASV level differential abundances between on- and off-tumor samples. For this analysis, we considered each of the three patients separately and used a linear model with location as a fixed effect, encoded as a binary categorical variable (on-/off-tumor). As shown in **Figure 2A**, ASVs that were strongly enriched or depleted (q-value threshold of 0.1 and absolute effect size of ≥2, corresponding to a four-fold difference in relative abundance) in tumor samples were affiliated with a broad range of genera. For patient B, ASVs that were significantly enriched and highly abundant in tumor samples (relative abundance of ≥0.5%, arithmetic mean across on-tumor samples) were taxonomically assigned to the genera *Leptotrichia* (asv183), *Streptococcus* (asv69), and *Fusobacterium* (asv105 and asv133) within SILVA’s taxonomic framework. For patient F, abundant tumor-enriched ASVs were assigned to more diverse genera, including *Filifactor* (asv154), *Fusobacterium* (asv65 and asv175), *Hungatella* (asv221), *Lentimicrobium* (asv206), *Leptotrichia* (asv271), *Porphyromonas* (asv239), *Treponema* (asv366 and asv521), and *Gemella* (asv78), within the top-10 most enriched ASVs (by the beta coefficient of the linear regression model). For patient G, only two ASVs assigned to the genera *Roseburia* (asv59) and *Treponema* (asv392) were enriched and highly abundant at the tumor site.

To strengthen the above findings, we further employed a linear mixed effects model to identify ASVs significantly enriched or depleted in on-tumor samples, considering data from all patients. Focusing on ASVs with an abundance of ≥0.01% in at least 10 samples (*n* = 477, 93.3 ± 8.0% of reads across samples), this revealed 12 ASVs that were significantly enriched in on-tumor samples (**Supplementary Figure S6**). These ASVs were affiliated with a range of genera, namely *Bacteroides* (asv19), *Campylobacter* (asv343), *Catonella* (asv311), *Centipeda* (asv518), *Fusobacterium* (asv175 and asv65), *Hungatella* (asv221), *Lachnospiraceae* NK4A136 group (asv261), *Parvimonas* (asv21), *Peptostreptococcus* (asv44), *Selenomonas* (asv201), *Treponema* (asv379). Only a single ASV was identified as depleted in on-tumor samples, namely asv224 affiliated with the genus *Actinomyces*.

A comparison of the ASVs against the Living Tree Project (LTP) database showed that several differentially abundant phylotypes represented *hitherto* uncultured species lacking validly described type strains (**Supplementary Table S3**). These included, for example, phylotypes asv206 (83.1% similarity to the 16S rRNA gene sequence of the type strain of *Perlabentimonas gracilis* in the LTP database, accession MT501785) and asv588 (93.7% similarity to *Leptotrichia trevisanii*, AF206305), both of which were enriched in the tumor of patient F.

The enrichment of specific ASVs in on-tumor samples tended to be highly localized and was no longer apparent even a few centimeters away from the tumors (**Figure 2B** and **Supplementary Figure S7**). For example, for patient B, a *Streptococcus*-related ASV (asv69; 100% similarity to *S. sanguinis*, accession AFAZ01000011; **Supplementary Table S3**) reached relative abundances exceeding 5% at the tumor site but had an abundance of only ~0.5% in surrounding noncancerous tissue, even within 1 cm from the tumor. Strong localization of specific ASVs at the tumor site was also observed in two other patients (F and G; **Supplementary Figure S7**). Similarly, depletion of specific ASVs tended to be localized at the tumor sites; however, for patient G, depletion of ASVs around the tumor was less pronounced, mirroring the trend in dissimilarities between the on- and off-tumor samples at the level of the entire community (**Figure 1B**).

In addition to the above-mentioned phylotypes, an inspection of all samples revealed additional abundant phylotypes distinct from type strains in the LTP database (**Supplementary Figure S8**). To enable better identification and understanding of these phylotypes, particularly phylotypes that may be involved in CRC carcinogenesis based on their high abundance in tumor tissue, we sought to acquire their draft genome sequences as MAGs. To this end, the microbiota in the tumor samples was enriched by short-term culturing under anaerobic conditions to reduce human DNA content before sequencing and subjected to shotgun metagenomics. MAGs were reconstructed by binning the assembled contigs and linked to ASVs generated by amplicon sequencing using MarkerMAG, a bioinformatics pipeline for linking 16S rRNA genes and MAGs, using paired-end sequencing reads (see Material and methods for details). Sequencing and metagenome assembly statistics are provided in **Supplementary Table S4**.

**Figure 3:**
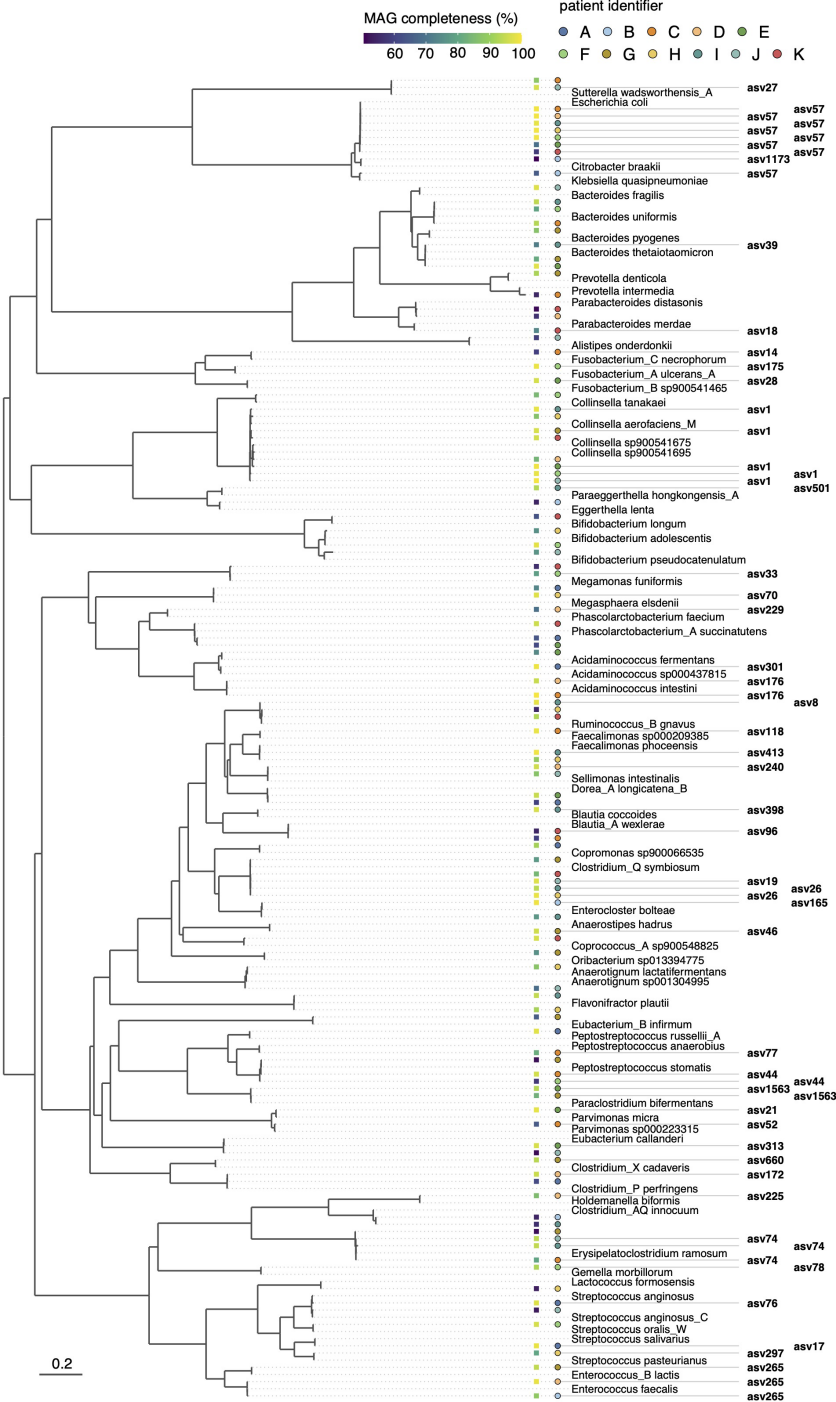
Phylogenetic bacterial genome tree of MAGs reconstructed from short-term enrichment cultures of colonic tumor tissue samples across all patients. The tree is based on the concatenated alignment of 120 single-copy marker gene proteins and was generated using GTDB-Tk. Representative GTDB genomes are shown for species with the same taxonomic assignment as the MAGs. Links to the ASVs, as inferred based on reconstructed 16S rRNA genes using MarkerMAG, are indicated. Filled squares adjacent to the tree show MAG completeness as estimated using CheckM, and filled circles indicate the patient from whom the MAG was derived. The scale bar indicates the number of amino acid substitutions per site.

In total, we obtained 115 medium-to-high-quality MAGs (completeness of ≥50% and contamination <5%, as estimated based on the presence of single-copy marker genes using CheckM), ranging from six MAGs for patient B to 13 MAGs for patients I and G (**Figure 3** and **Supplementary Table S5**). Taxonomic classification against the Genome Taxonomy Database (GTDB, release 207) showed that the reconstructed MAGs captured substantial phylogenetic diversity, including several MAGs representing genera/species previously linked to CRC. Using MarkerMAG, we were able to link 16S rRNA gene sequences for 65 MAGs (out of 115), 59 of which perfectly matched (100% sequence identity) the ASV generated using amplicon sequencing. For most MAG-ASV linkages, the taxonomic assignments of the MAGs and ASVs were consistent (**Supplementary Table S6**). Of note is that several species were represented by MAGs that were recovered in the enrichment cultures of multiple patients (**Supplementary Table S5**), including *Escherichia coli* (MAGs recovered in *n* = 7 enrichment cultures, out of 11), *Clostridium_Q symbiosum* (*n* = 5), unclassified MAGs within the genus *Collinsella* (*n* = 5), and *Erysipelatoclostridium ramosum* (*n* = 4).

Several ASV-linked MAGs represented genomes that were enriched at the tumor site compared with the surrounding non-tumor tissue (**Figure 3**). This included bin3 (linked to asv78), bin2 (asv44), and bin14 (asv175) in patient F. Within the GTDB taxonomic framework, these MAGs were classified as *Gemella morbillorum* (bin3, with 98.3% average nucleotide identity, ANI, to its closest representative genome in the GTDB, accession GCF_900476045.1, **Supplementary Table S5**), *Peptostreptococcus stomatis* (bin2, 97.9% ANI to GCF_000147675.1), and “Fusobacterium_A ulcerans_A” *(*bin14, 99.98% ANI to GCF_900683735.1). The reconstructed MAGs captured numerous taxa represented solely by uncultured microorganisms in the GTDB. Several of these, for example, “Fusobacterium_B sp900541465” for patient E and “Parvimonas sp000223315” for patient C, were relatively abundant as estimated based on their linked ASV abundances (**Supplementary Figure S9**).

## 4 Discussion

We profiled the mucosal microbiota in tumor and adjacent non-tumor tissues collected from patients with CRC who had undergone colonic resection without preoperative bowel preparation. Since bowel preparation has been shown to cause short-term perturbation of the gastrointestinal microbiota and changes in the observed microbiota profiles (Shobar et al., 2016), our study may thus provide a more representative depiction of the diversity and abundance of tissue-associated microbiota in patients with CRC. Furthermore, compared to previous studies, we further investigated the spatial heterogeneity of mucosal microbiota around the tumor site in greater detail by collecting samples at varying distances from the tumor with centimeter-scale resolution.

Within our cohort, community-wide microbiota dissimilarity between paired on- and off-tumor samples varied substantially among patients. This is broadly consistent with previous observations, with some studies reporting differences (Marchesi et al., 2011; Kostic et al., 2013; Boleij et al., 2015; Gao et al., 2015; Loke et al., 2018; Sheng et al., 2020; Zhu et al., 2022), whereas in other studies (Chen et al., 2012; Kostic et al., 2012; Flemer et al., 2017; Murphy et al., 2021) on- and off-tumor microbiota were highly similar. These observations suggest that patients may be grouped according to whether they harbor distinct tumor-associated microbiota compared with adjacent normal tissues. Within our dataset, such stratification did not appear to be correlated with patient characteristics, such as tumor-sidedness and cancer stage; future studies with a larger patient cohort are however required to substantiate this.

As a whole, we identified a range of bacteria with elevated abundance in on-tumor samples compared to patient-matched off-tumor samples, largely consistent with past studies (see e.g. Ternes et al., 2020 for review). This included ASVs affiliated with *Fusobacterium* (asv65 and asv175, linked to MAG bin14 for patient F), *Gemella morbillorum* (asv78, linked to bin3 for patient F), *Peptostreptococcus stomatis* (asv44, linked to bin9 and bin2 for patients C and F, respectively), *Parvimonas micra* (asv21, linked to bin9 for patient E), *Leptotrichia* (asv271), *Streptococcus* (asv69), *Selenomonas* (asv201), and *Treponema* (asv379). Several of these taxa, especially *Fusobacterium*, *Peptostreptococcus stomatis*, and *Parvimonas micra*, are frequently detected as enriched in colorectal adenoma and CRC patients, and the molecular mechanisms underlying their involvement in promoting colonic carcinogenesis are increasingly well understood (*e.g.*, Long et al., 2019; Chang et al., 2023). For others, such as *Leptotrichia* (Xu and Jiang, 2017), *Gemella morbillorum* (Avuthu and Guda, 2022) and *Treponema* (Yang et al., 2019), past studies have observed an association with the risk of CRC or increased abundance in patients, but the molecular processes through which they affect CRC are less well understood. In particular, it remains to be resolved whether the bacterial taxa identified as enriched in tumor tissue initiate and drive carcinogenesis or act as “passengers” that take advantage of the tumor microenvironment to outcompete other bacteria (Avril and DePaolo, 2021). Of note is here that the dense sampling of some patients revealed that the enrichment of these bacteria was highly localized to the tumor and disappeared within a few centimeters of the tumor.

Within the genus *Fusobacterium*, multiple phylotypes distinct from *F. nucleatum*, the main *Fusobacterium* species previously linked to CRC, were strongly enriched at the tumor site. The MAG of “Fusobacterium_A ulcerans_A” recovered from patient F was predicted to encode for FadA adhesin (**Supplementary Table S7**), a well-established virulence factor associated with CRC (Rubinstein et al., 2013). In line with this, Yeoh et al. (2020) recently reported that several fusobacterial lineages distinct from *F. nucleatum*, including *F. ulcerans* within the “Fusobacterium_A” clade within the GTDB, possessed FadA homologues in Southern Chinese populations.

We further found that several species in the enrichment cultures constructed from on-tumor tissue samples were represented by MAGs recovered from multiple patients. In addition to *E. coli*, this included MAGs affiliated with the species *E. ramosum*, *C. symbiosum*, and unclassified MAGs within the genus *Collinsella*. Although not as commonly associated with CRC, a recent study by Iadsee et al. (2023) found that *E. ramosum* was enriched in mucosal and luminal microbiota of Thai CRC patients, and suggested that this species may represent a tumor-promoting bacterium that preferentially colonizes the tumor microenvironment and present a biomarker for CRC screening. For *C. symbiosum*, several past studies observed a stepwise increase in the abundance of this species in the feces of healthy controls, colorectal adenoma, and CRC patients (Xie et al., 2017; Zhang et al., 2021) and *C. symbiosum* was also identified as a CRC-associated bacterium in a meta-analysis of cohorts from diverse geographical regions (Avuthu and Guda, 2022).

Our study has a number of limitations to be recognized. Firstly, due to the fact that preoperative bowel cleansing before colorectal surgery has become routine clinical practice worldwide, only a relatively small number of patients could be recruited. This also means that colonic tissue collection during surgery from unprepped patients may be challenging to adopt at larger scales. Secondly, mucosal microbiota and their differences between on- and off-tumor microbiota were highly variable between patients, and larger sample sizes will be needed to improve statistical power. Thirdly, only a limited number of on-tumor samples were collected for each patient; more extensive sampling of tumor-associated microbiota will be needed to better account for potential within-tumor heterogeneity of microbiota profiles.

This study also underscored the need to continue to acquire more detailed information on bacterial populations linked to CRC through cultivation and/or genome sequencing. Herein, we acquired MAGs of several tumor-associated bacteria and a range of uncultured species. The availability of representative genome sequences is needed to clarify microbial features, such as the production of toxins, which may be responsible for their association with CRC and will also facilitate efforts to cultivate them.

## Data availability statement

The datasets presented in this study can be found in online repositories. The names of the repository/repositories and accession number(s) can be found in the article/**Supplementary Material**.

## Ethics statement

The studies involving human participants were reviewed and approved by Ethics Committee of Yokohama City University (B190600051, F220600030). The patients/participants provided their written informed consent to participate in this study.

## Author contributions

HF performed sampling, data analysis, and prepared the manuscript. DT performed data analysis and prepared the manuscript. AO performed laboratory experiments, including DNA extraction, culturing, and DNA sequencing. SS, KN, MO, and AI performed surgery and sampling. IE performed sampling and prepared the manuscript. YS performed data analysis and prepared the manuscript. All authors have reviewed and approved the submitted manuscript.
