# Supplementary material for: Elucidating colorectal cancer-associated bacteria through profiling of minimally perturbed tissue-associated microbiota
Source: Front Cell Infect Microbiol. 2023 Aug 1;13:1216024. doi: 10.3389/fcimb.2023.1216024 (PMC10432157; doi:10.3389/fcimb.2023.1216024)
Supplement: Supplementary file 1 [file DataSheet_1.docx]

Supplementary Material

Elucidating colorectal cancer-associated bacteria through profiling of minimally perturbed tissue-associated microbiota

Hironori Fukuoka, Dieter M. Tourlousse, Akiko Ohashi, Shinsuke Suzuki, Kazuya Nakagawa, Mayumi Ozawa, Atsushi Ishibe, Itaru Endo, Yuji Sekiguchi^*^

*** Correspondence:** Yuji Sekiguchi [y.sekiguchi@aist.go.jp](mailto:y.sekiguchi@aist.go.jp)

**Supplementary Tables**

**Supplementary Table S1.** Characteristics of patients included in this study.

| **Patient**  **identifier** | **Age**  **(years)** | **Sex** | **Body mass index**  **(kg m^-2^)** | **Smoking**  **status** | **Medical**  **comorbidities ^a^** | **Past**  **gastroenterological**  **surgery** | **Tumor**  **location** | **T** | **N** | **M** | **Tumor stage**  **(TNM) ^b^** | **Histological findings ^c^** | **Pre-operative**  **antibiotics use** | **Pre-operative**  **PPI use ^e^** | **Operative**  **procedure ^f^** |
| --- | --- | --- | --- | --- | --- | --- | --- | --- | --- | --- | --- | --- | --- | --- | --- |
| A | 73 | female | 23.2 | none | HT, DM | none | ascending colon | 3 | 0 | 0 | IIA | moderately | no | no | lap-RHC |
| B | 77 | male | 20.7 | none | rectal cancer | anterior resection | cecum | 2 | 0 | 0 | I | moderately | no | no | lap-ICR |
| C | 68 | male | 19.2 | none | HT, asthma | none | sigmoid colon | 3 | 0 | 0 | IIA | moderately | no | no | lap-S |
| D | 75 | male | 23.8 | current | HT, DM, HLP | appendectomy | sigmoid colon | 4a | 1a | 0 | IIIB | well | yes ^d^ | yes | lap-S |
| E | 69 | male | 28.0 | past | CI | none | sigmoid colon | 3 | 0 | 0 | IIA | moderately | no | yes | lap-S |
| F | 52 | female | 22.5 | none | HT, WPW syndrome | none | sigmoid colon | 4a | 1b | 0 | IIIB | moderately | no | no | lap-S |
| G | 70 | female | 19.6 | none | none | none | ascending colon | 4b | 1a | 0 | IIIC | NEC | no | no | lap-RHC |
| H | 77 | male | 24.4 | past | HT, DM, HU | none | transverse colon | 3 | 2a | 0 | IIIB | moderately | no | no | lap-RHC |
| I | 79 | male | 25.3 | past | HT, DM | none | ascending colon | 3 | 2b | 0 | IIIC | well | no | no | lap-RHC |
| J | 71 | male | 22.0 | none | none | appendectomy | rectum | 3 | 1b | 0 | IIIB | moderately | no | no | lap-LAR |
| K | 71 | male | 22.4 | past | none | appendectomy | ascending colon | 3 | 1b | 0 | IIIB | moderately | no | no | lap-RHC |

^a^ HT: hypertension; DM: diabetes mellitus; HU: hyperuricemia; HLP: hyperlipidemia; CI: cerebral infarction; WPW: Wolff-Parkinson-White syndrome.

^b^ Cancer Stage was determined according to the Union for International Cancer Control (UICC) TNM Classification of Malignant Tumours.

^c^ moderately: moderately differentiated adenocarcinoma, well: well-differentiated adenocarcinoma, NEC: neuroendocrine carcinoma.

^d^ Oral metronidazole administration one day prior to surgery.

^e^ PPI: proton-pump inhibitors.

^f^ lap: laparoscopy; RHC: right-hemicolectomy; S: sigmoidectomy; LA: low anterior resection; ICR: ileocecal resection.

**Supplementary Table S2.** Statistics of the 16S rRNA gene amplicon sequencing data generated in this study.

| **Patient**  **identifier** | **Accession**  **number** | **Sample**  **location** | **Reads**  **raw** | **Reads**  **Cutadapt** | **Reads**  **FilterAndTrim** | **Reads**  **DADA2** | **Reads**  **DADA2, filtered** | **ASVs**  **DADA2** | **ASVs**  **DADA2, filtered** |
| --- | --- | --- | --- | --- | --- | --- | --- | --- | --- |
|  |  |  |  |  |  |  |  |  |  |
| A | SRX17146378 | -10 | 104,014 | 101,324 | 100,842 | 95,197 | 95,006 | 302 | 298 |
| A | SRX17146389 | -10 | 85,266 | 82,824 | 82,399 | 75,094 | 74,950 | 294 | 289 |
| A | SRX17146309 | -9 | 38,769 | 38,010 | 37,828 | 35,714 | 35,705 | 242 | 239 |
| A | SRX17146308 | -8 | 42,458 | 41,690 | 41,498 | 39,301 | 39,292 | 244 | 242 |
| A | SRX17146307 | -7 | 40,938 | 40,086 | 39,892 | 38,086 | 38,078 | 244 | 242 |
| A | SRX17146306 | -6 | 47,632 | 46,613 | 46,427 | 43,792 | 43,708 | 251 | 248 |
| A | SRX17146304 | -5 | 95,360 | 92,811 | 92,310 | 85,333 | 85,082 | 236 | 232 |
| A | SRX17146305 | -5 | 42,727 | 41,891 | 41,733 | 39,656 | 39,578 | 228 | 226 |
| A | SRX17146362 | -4 | 30,178 | 29,551 | 29,413 | 27,291 | 27,287 | 192 | 191 |
| A | SRX17146351 | -3 | 36,684 | 35,983 | 35,814 | 33,749 | 33,746 | 239 | 238 |
| A | SRX17146340 | -2 | 31,180 | 30,532 | 30,438 | 28,438 | 28,427 | 231 | 229 |
| A | SRX17146367 | -1 | 95,453 | 92,408 | 91,844 | 84,758 | 84,621 | 325 | 318 |
| A | SRX17146302 | 0 | 94,841 | 92,407 | 91,963 | 87,413 | 87,265 | 314 | 310 |
| A | SRX17146303 | 0 | 86,053 | 83,895 | 83,483 | 78,893 | 78,746 | 311 | 307 |
| A | SRX17146325 | 0 | 81,491 | 78,945 | 78,519 | 73,847 | 73,807 | 303 | 297 |
| A | SRX17146314 | 0 | 90,012 | 87,353 | 86,909 | 81,035 | 80,883 | 310 | 304 |
| A | SRX17146310 | 1 | 70,020 | 67,616 | 67,148 | 61,925 | 61,800 | 263 | 259 |
| A | SRX17146313 | 2 | 102,746 | 99,713 | 99,185 | 91,783 | 91,567 | 302 | 299 |
| A | SRX17146315 | 3 | 110,241 | 106,989 | 106,344 | 99,717 | 99,538 | 315 | 310 |
| A | SRX17146316 | 4 | 96,168 | 93,137 | 92,660 | 86,433 | 86,238 | 313 | 309 |
| A | SRX17146317 | 5 | 107,458 | 104,807 | 104,202 | 98,477 | 98,269 | 352 | 339 |
| A | SRX17146318 | 5 | 98,236 | 95,275 | 94,702 | 88,437 | 88,244 | 330 | 325 |
| A | SRX17146319 | 6 | 81,070 | 78,507 | 78,009 | 72,133 | 71,983 | 287 | 281 |
| A | SRX17146320 | 7 | 124,081 | 121,356 | 120,925 | 112,414 | 112,129 | 315 | 308 |
| A | SRX17146321 | 8 | 114,760 | 112,148 | 111,723 | 104,369 | 104,155 | 309 | 305 |
| A | SRX17146322 | 9 | 80,380 | 78,203 | 77,804 | 71,274 | 71,122 | 276 | 271 |
| A | SRX17146311 | 10 | 64,264 | 62,378 | 61,993 | 57,904 | 57,787 | 255 | 254 |
| A | SRX17146312 | 10 | 149,423 | 145,967 | 145,442 | 135,716 | 135,438 | 348 | 343 |
| B | SRX17146327 | -10 | 139,387 | 131,053 | 130,263 | 121,296 | 121,296 | 110 | 110 |
| B | SRX17146366 | -9 | 151,029 | 142,155 | 141,284 | 131,819 | 131,819 | 129 | 129 |
| B | SRX17146334 | -8 | 93,503 | 87,727 | 87,056 | 80,581 | 80,581 | 110 | 110 |
| B | SRX17146333 | -7 | 110,939 | 104,286 | 103,578 | 95,919 | 95,919 | 119 | 119 |
| B | SRX17146332 | -6 | 98,344 | 92,395 | 91,832 | 84,515 | 84,515 | 104 | 104 |
| B | SRX17146331 | -5 | 154,286 | 145,260 | 144,309 | 133,403 | 133,403 | 130 | 130 |
| B | SRX17146330 | -4 | 99,288 | 93,816 | 93,166 | 85,335 | 85,335 | 120 | 120 |
| B | SRX17146329 | -3 | 88,867 | 83,936 | 83,335 | 75,901 | 75,901 | 100 | 100 |
| B | SRX17146328 | -2 | 152,190 | 143,911 | 142,836 | 128,007 | 128,007 | 120 | 120 |
| B | SRX17146326 | -1 | 141,611 | 133,918 | 133,028 | 116,861 | 116,861 | 113 | 113 |
| B | SRX17146323 | 0 | 111,426 | 104,974 | 104,386 | 95,015 | 95,015 | 106 | 106 |
| B | SRX17146324 | 0 | 113,583 | 106,934 | 106,281 | 97,557 | 97,557 | 111 | 111 |
| B | SRX17146368 | 1 | 95,688 | 90,271 | 89,629 | 81,299 | 81,299 | 106 | 106 |
| B | SRX17146370 | 2 | 110,900 | 104,816 | 104,163 | 95,259 | 95,259 | 109 | 109 |
| B | SRX17146371 | 3 | 112,545 | 106,178 | 105,386 | 96,215 | 96,215 | 120 | 120 |
| B | SRX17146372 | 4 | 113,195 | 106,932 | 106,232 | 98,314 | 98,314 | 114 | 114 |
| B | SRX17146373 | 5 | 133,306 | 125,525 | 124,568 | 113,573 | 113,573 | 124 | 124 |
| B | SRX17146374 | 6 | 105,563 | 99,336 | 98,492 | 89,566 | 89,566 | 118 | 118 |
| B | SRX17146375 | 7 | 120,051 | 113,047 | 112,169 | 99,255 | 99,255 | 122 | 122 |
| B | SRX17146376 | 8 | 121,491 | 114,466 | 113,585 | 103,125 | 103,125 | 122 | 122 |
| B | SRX17146377 | 9 | 87,792 | 82,285 | 81,548 | 73,755 | 73,755 | 104 | 104 |
| B | SRX17146369 | 10 | 103,787 | 97,862 | 97,006 | 86,027 | 86,027 | 113 | 113 |
| C | SRX17146387 | -9 | 84,144 | 82,414 | 82,174 | 77,027 | 77,000 | 224 | 222 |
| C | SRX17146386 | -8 | 59,622 | 58,191 | 57,981 | 54,917 | 54,917 | 176 | 176 |
| C | SRX17146385 | -7 | 116,835 | 114,250 | 113,865 | 109,152 | 109,119 | 222 | 218 |
| C | SRX17146384 | -6 | 121,936 | 119,235 | 118,959 | 113,846 | 113,812 | 216 | 213 |
| C | SRX17146383 | -4 | 101,923 | 99,661 | 99,330 | 95,466 | 95,440 | 226 | 223 |
| C | SRX17146382 | -3 | 87,431 | 85,483 | 85,220 | 81,487 | 81,473 | 205 | 203 |
| C | SRX17146381 | -2 | 98,910 | 96,289 | 95,941 | 91,414 | 91,409 | 211 | 210 |
| C | SRX17146379 | 0 | 75,083 | 72,993 | 72,643 | 69,316 | 69,299 | 196 | 195 |
| C | SRX17146380 | 0 | 110,447 | 107,387 | 106,945 | 102,604 | 102,576 | 224 | 222 |
| D | SRX17146392 | -7 | 91,392 | 89,341 | 89,067 | 78,328 | 78,304 | 180 | 176 |
| D | SRX17146391 | -2 | 52,755 | 51,263 | 51,064 | 43,990 | 43,968 | 159 | 155 |
| D | SRX17146388 | 0 | 66,175 | 63,461 | 62,952 | 52,677 | 52,668 | 143 | 142 |
| D | SRX17146390 | 0 | 83,169 | 81,144 | 80,924 | 74,653 | 74,646 | 183 | 182 |
| D | SRX17146393 | 2 | 92,068 | 89,579 | 89,218 | 77,206 | 77,197 | 176 | 175 |
| D | SRX17146394 | 7 | 93,101 | 90,947 | 90,682 | 76,363 | 76,338 | 188 | 184 |
| E | SRX17146337 | -10 | 111,578 | 108,750 | 108,457 | 102,705 | 102,705 | 168 | 168 |
| E | SRX17146338 | -5 | 96,030 | 93,567 | 93,309 | 87,795 | 87,795 | 158 | 158 |
| E | SRX17146335 | 0 | 80,571 | 78,560 | 78,337 | 74,639 | 74,633 | 170 | 169 |
| E | SRX17146336 | 0 | 95,926 | 93,508 | 93,212 | 89,205 | 89,205 | 166 | 166 |
| E | SRX17146341 | 5 | 69,046 | 67,169 | 66,924 | 63,783 | 63,783 | 148 | 148 |
| E | SRX17146339 | 10 | 115,545 | 112,710 | 112,380 | 105,316 | 105,316 | 185 | 185 |
| F | SRX17146344 | -10 | 117,436 | 114,492 | 113,874 | 103,836 | 103,384 | 326 | 315 |
| F | SRX17146345 | -5 | 94,122 | 91,882 | 91,494 | 84,743 | 84,323 | 314 | 301 |
| F | SRX17146342 | 0 | 89,682 | 87,618 | 87,339 | 82,587 | 82,405 | 277 | 269 |
| F | SRX17146343 | 0 | 67,667 | 65,785 | 65,602 | 61,090 | 61,013 | 224 | 217 |
| F | SRX17146347 | 5 | 92,031 | 89,761 | 89,375 | 82,362 | 81,946 | 296 | 284 |
| F | SRX17146346 | 10 | 100,148 | 97,860 | 97,460 | 89,760 | 89,355 | 309 | 298 |
| G | SRX17146350 | -10 | 125,098 | 121,892 | 121,269 | 99,471 | 99,467 | 242 | 241 |
| G | SRX17146352 | -5 | 102,563 | 100,073 | 99,573 | 92,684 | 92,658 | 255 | 254 |
| G | SRX17146349 | 0 | 102,017 | 99,045 | 98,581 | 90,496 | 90,440 | 228 | 225 |
| G | SRX17146348 | 0 | 79,100 | 77,276 | 76,821 | 67,115 | 67,112 | 210 | 209 |
| G | SRX17146354 | 5 | 117,774 | 114,905 | 114,406 | 106,330 | 106,174 | 244 | 242 |
| G | SRX17146353 | 10 | 125,096 | 121,785 | 120,923 | 115,863 | 115,815 | 187 | 186 |
| H | SRX17146357 | -10 | 121,598 | 118,903 | 118,584 | 104,767 | 102,866 | 164 | 163 |
| H | SRX17146358 | -5 | 66,785 | 65,034 | 64,754 | 58,926 | 57,967 | 137 | 136 |
| H | SRX17146355 | 0 | 109,907 | 107,421 | 107,112 | 96,883 | 95,798 | 181 | 178 |
| H | SRX17146356 | 0 | 167,531 | 163,407 | 162,872 | 151,775 | 150,150 | 201 | 198 |
| I | SRX17146361 | -10 | 102,526 | 100,267 | 99,904 | 87,147 | 87,089 | 255 | 247 |
| I | SRX17146363 | -5 | 93,272 | 91,030 | 90,641 | 79,897 | 79,770 | 256 | 244 |
| I | SRX17146359 | 0 | 67,643 | 65,355 | 65,048 | 60,915 | 60,814 | 235 | 230 |
| I | SRX17146360 | 0 | 81,179 | 78,589 | 78,218 | 71,850 | 71,679 | 250 | 239 |
| J | SRX17146395 | -10 | 104,893 | 101,608 | 101,227 | 92,520 | 92,025 | 297 | 284 |
| J | SRX17146396 | -5 | 114,380 | 110,616 | 110,114 | 103,848 | 103,232 | 313 | 297 |
| J | SRX17146365 | 0 | 73,517 | 70,939 | 70,604 | 66,016 | 65,630 | 270 | 259 |
| J | SRX17146364 | 0 | 113,339 | 109,843 | 109,644 | 102,498 | 102,383 | 216 | 207 |
| K | SRX17146398 | -5 | 220,001 | 212,457 | 202,555 | 168,470 | 166,939 | 113 | 111 |
| K | SRX17146397 | 0 | 284,064 | 273,430 | 259,326 | 221,486 | 220,267 | 131 | 129 |
| K | SRX17146399 | 5 | 155,056 | 149,742 | 141,478 | 122,682 | 121,757 | 142 | 140 |

Sample location: distance to the tumor (in cm), with negative and positive values indicating locations toward the oral and anal side of the colon, respectively.

Reads raw: number of sequenced read pairs.

Reads Cutadapt: number of read pairs retained after processing by Cutadapt.

Reads FilterAndTrim: number of read pairs retained after processing by DADA2’s FilterAndTrim.

Reads DADA2: total number of reads in DADA2’s count table, that is, after denoising, merging, and removal of bimeras.

ASVs DADA2: total number of ASVs in DADA2’s count table, that is, after denoising, merging, and removal of bimeras.

Reads DADA2, filtered: total number of reads in DADA2’s count table after elimination of spurious ASVs, based on their length and taxonomic assignment (see Methods for details).

ASVs DADA2, filtered: total number of ASVs in DADA2’s count table after elimination of spurious ASVs, based on their length and taxonomic assignment (see Methods for details).

**Supplementary Table S3.** Closest relatives in the LTP database for phylotypes/ASVs plotted in Fig. 2a.

| **ASV identifier** | **Genus-level taxonomic classification**  **(Silva database, release 138) ^a^** | **Best-match within LTP database (release LTP_01_2022)**  **Organism name [accession number, similarity/identity] ^b^** |
| --- | --- | --- |
| asv105 | Fusobacterium | Fusobacterium canifelinum [AY162221, 100%] |
| asv1096 | Family XIII AD3011 group | Aminipila butyrica [AB298771, 95.2%] |
|  |  | Anaerovorax odorimutans [AJ251215, 95.2%] |
| asv113 | [Eubacterium] hallii group | Anaerobutyricum soehngenii [AJ270490, 99.6%] |
| asv124 | Blautia | Blautia caecimuris [KR364746, 100%] |
| asv133 | Fusobacterium | Fusobacterium nucleatum subsp. polymorphum [AF287812, 99.6%]  F. canifelinum [AY162221, 99.6%] |
| asv142 | Ruminococcus | Ruminococcus champanellensis [AB910742, 98%] |
| asv154 | Filifactor | Filifactor villosus [AF537211, 96.4%] |
| asv16 | Bifidobacterium | Bifidobacterium breve [AB006658, 100%]  B. longum subsp. suillum [AB924532, 100%]  B. longum subsp. infantis [D86184, 100%]  B. olomucense [MN707966, 100%],  B. scaligerum [PGLQ01000000, 100%] |
| asv175 | Fusobacterium | Fusobacterium varium [AJ867036, 99.2%] |
| asv181 | Solobacterium | Solobacterium moorei [AB540987, 100%] |
| asv183 | Leptotrichia | Leptotrichia hongkongensis [EU919515, 100%] |
| asv19 | Bacteroides | Bacteroides fragilis [CR626927, 100%] |
| asv206 | Lentimicrobium | Perlabentimonas gracilis [MT501785, 83.1%] |
| asv21 | Parvimonas | Parvimonas micra [AY323523, 100%] |
| asv211 | Fusobacterium | Fusobacterium watanabei [LC514065, 100%] |
| asv216 | Bacteroides | Bacteroides salyersiae [AQHX01000008, 99.6%] |
| asv219 | un. Veillonellaceae | Dialister pneumosintes [X82500, 100%] |
| asv221 | Hungatella | Hungatella effluvii [HE603919, 100%] |
| asv239 | Porphyromonas | Porphyromonas somerae [AB547667, 100%] |
| asv240 | Sellimonas | Sellimonas intestinalis [KP966092, 100%] |
| asv244 | Frisingicoccus | Frisingicoccus caecimuris [KR364774, 98.4%] |
| asv26 | un. Lachnospiraceae | Clostridium nexile [X73443, 99.2%] |
| asv261 | Lachnospiraceae NK4A136 group | Enterocloster aldenensis [DQ279736, 99.6%] |
| asv267 | Alistipes | Alistipes communis [LC468799, 100%] |
| asv271 | Leptotrichia | Leptotrichia hofstadii [AB558170, 98.4%] |
| asv281 | un. Lachnospiraceae | Clostridium hylemonae [AB910737, 100%] |
| asv312 | Lachnospiraceae UCG-009 | Enterocloster asparagiformis [ACCJ01000522, 100%]  Enterocloster lavalensis [EF564277, 100%] |
| asv316 | Porphyromonas | Porphyromonas asaccharolytica [CP002689, 99.6%] |
| asv325 | un. Lachnospiraceae | Anaerotignum aminivorans [AB298756, 94.5%] |
| asv33 | Megamonas | Megamonas funiformis [ADMB01000074, 100%] |
| asv343 | Campylobacter | Campylobacter rectus [AB595133, 100%]  C. showae [DQ174155, 100%]  C. massiliensis [MT882021, 100%] |
| asv366 | Treponema | Treponema medium [ATFE01000011, 98.8%] |
| asv368 | Paraprevotella | Paraprevotella clara [AB547651, 97.2%] |
| asv37 | Agathobacter | Agathobacter rectalis [CP001107, 100%] |
| asv379 | Treponema | Treponema maltophilum [ATFF01000006, 99.6%] |
| asv38 | Bifidobacterium | Bifidobacterium adolescentis [AP009256, 100%]  B. faecale [KF990498, 100%] |
| asv392 | Treponema | Treponema socranskii subsp. socranskii [AF033306, 100%] |
| asv44 | Peptostreptococcus | Peptostreptococcus stomatis [DQ160208, 100%] |
| asv453 | Sellimonas | Sellimonas monacensis [MN055941, 100%] |
| asv459 | Frisingicoccus | Frisingicoccus caecimuris [KR364774, 98%] |
| asv46 | Anaerostipes | Anaerostipes hadrus [FR749932, 100%] |
| asv47 | Fusobacterium | Fusobacterium nucleatum subsp. vincentii [AABF01000111, 100%]  F. naviforme [AJ006965, 100%] |
| asv50 | Alistipes | Alistipes putredinis [ABFK02000016, 100%] |
| asv502 | Slackia | Slackia exigua [ACUX02000005, 100%] |
| asv506 | Bergeyella | Spodiobacter cordis [AB818539, 98.4%] |
| asv521 | Treponema | Treponema socranskii subsp. socranskii [AF033306, 99.6%] |
| asv524 | Paraprevotella | Paraprevotella clara [AB547651, 99.2%] |
| asv55 | Sutterella | Sutterella wadsworthensis [HM037997, 100%] |
| asv564 | Blautia | Blautia hydrogenotrophica [ACBZ01000217, 96.4%] |
| asv568 | Alistipes | Alistipes inops [JRGF01000044, 100%] |
| asv575 | Alistipes | Alistipes ihumii [JX101692, 100%] |
| asv58 | Lachnospiraceae UCG-010 | Anaerotignum faecicola [MK439508, 99.6%] |
| asv588 | Leptotrichia | Leptotrichia trevisanii [AF206305, 93.7%] |
| asv59 | Roseburia | Roseburia inulinivorans [AJ270473, 100%] |
| asv592 | Erysipelatoclostridium | Longibaculum muris [KR364765, 92.9%] |
| asv60 | Blautia | Blautia obeum [X85101, 97.2%] |
| asv606 | Marvinbryantia | Blautia wexlerae [EF036467, 96.4%] |
| asv621 | Veillonella | Veillonella dispar [ACIK02000021, 100%] |
| asv631 | Centipeda | Selenomonas infelix [AF287802, 99.6%]  Selenomonas timonae [MT764255, 99.6%] |
| asv633 | Blautia | Blautia obeum [X85101, 96.8%] |
| asv65 | Fusobacterium | Fusobacterium nucleatum subsp. animalis [AFQD01000460, 100%] |
| asv67 | Streptococcus | Streptococcus oralis oralis subsp. oralis [ADMV01000001, 100%]  S. infantis [AEVD01000030, 100%], S. mitis [AF003929, 100%]  S. oralis subsp. tigurinus [AORU01000002, 100%]  S. oralis subsp. dentisani [HG315101, 100%]  S. downii [MF443455, 100%] |
| asv670 | Shuttleworthia | Roseburia faecis [AY305310, 95.3%] |
| asv678 | Intestinimonas | Intestinimonas butyriciproducens [KC311367, 97.6%] |
| asv679 | Centipeda | Selenomonas infelix [AF287802, 99.2%]  S. [MT764255, 99.2%] |
| asv69 | Streptococcus | Streptococcus sanguinis [AFAZ01000011, 100%] |
| asv692 | un. Barnesiellaceae | Coprobacter fastidiosus [JN703378, 90.9%] |
| asv750 | Incertae Sedis | Acutalibacter muris [KR364749, 96.8%] |
| asv76 | Streptococcus | Streptococcus anginosus subsp. anginosus [AFIM01000033, 100%]  S. anginosus subsp. whileyi [JN787193, 100%] |
| asv78 | Gemella | Gemella parahaemolysans [HM103931, 100%]  G. taiwanensis [HM103934, 100%]  G. haemolysans [L14326, 100%]  G. morbillorum [L14327, 100%]  G. sanguinis [Y13364, 100%] |
| asv808 | Marvinbryantia | Blautia faecis [HM626178, 97.6%] |
| asv814 | un. Lachnospiraceae | Blautia argi [KX025138, 97.6%] |
| asv837 | Mogibacterium | Mogibacterium neglectum [AB037875, 99.6%]  M. pumilum [CP016199, 99.6%] |
| asv844 | Prevotella | Prevotella nigrescens [AFPX01000069, 100%] |
| asv859 | Intestinimonas | Pseudoflavonifractor capillosus [AY136666, 97.2%] |

^a^ Assigned against the Silva database (file "silva_nr99_v138.1_train_set.fa.gz") using DADA2’s assignTaxonomy. “un.” indicates unclassified (minimum bootstrap confidence of 50).

^b^ Search against LTP database (file "LTP_01_2022_compressed.fasta"). All best matches based on sequence identity are listed for each ASV.

**Supplementary Table S4.** Statistics of the shotgun metagenome sequencing data generated in this study.

| **Patient**  **identifier** | **Accession**  **number ^a^** | **Reads pairs,**  **raw** | **Read pairs,**  **after fastp** | **Read pairs,**  **after BMTagger ^a^** | **Assembly**  **length (bp) ^b^** | **Assembly**  **no. of contigs ^b^** | **Assembly**  **N50 ^b^** |
| --- | --- | --- | --- | --- | --- | --- | --- |
| A | SRX17171584 | 6,268,362 | 5,665,697 | 5,665,499 | 81,967,994 | 18,885 | 5,551 |
| B | SRX17171585 | 7,438,340 | 6,190,783 | 6,190,749 | 34,277,415 | 3,058 | 34,046 |
| C | SRX17171587 | 6,321,881 | 4,836,958 | 4,835,400 | 61,465,120 | 12,586 | 7,686 |
| D | SRX17171588 | 9,024,532 | 8,162,930 | 8,161,240 | 69,332,723 | 12,540 | 10,915 |
| E | SRX17171589 | 6,621,447 | 6,066,000 | 6,061,242 | 64,283,237 | 12,210 | 9,865 |
| F | SRX17171590 | 5,671,619 | 5,187,154 | 5,181,741 | 69,532,215 | 10,038 | 14,571 |
| G | SRX17171591 | 6,925,352 | 6,354,493 | 6,353,390 | 77,449,419 | 15,284 | 8,297 |
| H | SRX17171592 | 6,274,483 | 5,705,108 | 5,703,116 | 68,525,580 | 11,216 | 11,117 |
| I | SRX17171593 | 7,211,847 | 6,576,070 | 6,548,552 | 73,306,150 | 10,042 | 19,861 |
| J | SRX17171594 | 5,896,255 | 5,240,200 | 5,235,682 | 66,599,225 | 14,077 | 6,870 |
| K | SRX17171586 | 6,770,322 | 6,185,889 | 6,181,256 | 69,273,117 | 11,218 | 12,195 |

^a^ Sequence data available in NCBI’s SRA database represents reads after processing with fastp and BMTagger.

^b^ Calculated for contigs with a minimum length of 1,500 bp.

**Supplementary Table S5.** Summary of MAGs reconstructed from tumor tissue samples after short-term enrichment culturing.

| **Patient identifier** | **MAG identifier** | **MAG classification ^a^** | **closest**  **placement**  **reference ^b^** | **closest**  **placemen**  **taxonomy ^b^** | **closest**  **placement**  **ANI ^b^** | **closest**  **placement**  **AF ^b^** | **Length, bp** | **Contigs, no.** | **N50, bp** | **Completeness ^c^, %** | **Contamination ^c^, %** | **GC content, %**  **n** | **CDSs ^d^, no.** | **rRNAs ^d^, no.** | **tRNAs ^d^, no.** |
| --- | --- | --- | --- | --- | --- | --- | --- | --- | --- | --- | --- | --- | --- | --- | --- |
|  |  |  |  |  |  |  |  |  |  |  |  |  |  |  |  |
| A | bin8 | Clostridium_P perfringens | GCF_000013285.1 | s__Clostridium_P perfringens | 98.21 | 0.90 | 2,500,570 | 57 | 75,542 | 62.1 | 1.7 | 27.8 | 2239 | 1 | 18 |
| A | bin4 | Streptococcus anginosus | GCF_900636475.1 | s__Streptococcus anginosus | 96.59 | 0.94 | 1,717,728 | 45 | 74,941 | 97.4 | 0.0 | 39.1 | 1648 | 0 | 22 |
| A | bin2 | Streptococcus salivarius | N/A | N/A | N/A | N/A | 2,134,268 | 56 | 64,520 | 98.6 | 0.2 | 39.8 | 1948 | 1 | 27 |
| A | bin25 | Acidaminococcus sp000437815 | GCF_009696675.1 | s__Acidaminococcus sp000437815 | 97.54 | 0.93 | 2,035,460 | 82 | 32,147 | 99.1 | 0.6 | 56.6 | 1855 | 1 | 56 |
| A | bin12 | Peptostreptococcus russellii_A | GCF_900110295.1 | s__Peptostreptococcus russellii_A | 99.20 | 0.91 | 2,044,674 | 159 | 20,817 | 97.9 | 0.9 | 30.7 | 1682 | 1 | 23 |
| A | bin3 | Dorea_A longicatena_B | GCF_001404875.1 | s__Dorea_A longicatena_B | 97.75 | 0.90 | 1,296,613 | 100 | 17,155 | 59.7 | 0.0 | 42.6 | 1131 | 0 | 16 |
| A | bin1 | Copromonas sp900066535 | GCF_003435375.1 | s__Copromonas sp900066535 | 97.97 | 0.90 | 2,537,010 | 217 | 16,375 | 91.5 | 1.3 | 49.5 | 2119 | 0 | 46 |
| A | bin18 | Megasphaera elsdenii | GCF_003010495.1 | s__Megasphaera elsdenii | 98.30 | 0.98 | 1,633,967 | 400 | 4,757 | 73.6 | 0.3 | 54.4 | 1240 | 1 | 40 |
| A | bin22 | Phascolarctobacterium_A succinatutens | GCF_000188175.1 | s__Phascolarctobacterium_A succinatutens | 99.14 | 0.98 | 1,410,236 | 387 | 4,089 | 64.4 | 1.8 | 48.4 | 1045 | 0 | 28 |
| B | bin5 | Clostridium_AQ innocuum | GCF_012317185.1 | s__Clostridium_AQ innocuum | 97.41 | 0.87 | 2,023,443 | 21 | 189,506 | 54.4 | 0.0 | 44.6 | 1961 | 1 | 29 |
| B | bin10 | Enterococcus faecalis | GCF_000392875.1 | s__Enterococcus faecalis | 98.61 | 0.88 | 2,561,326 | 25 | 182,085 | 88.4 | 0.4 | 37.6 | 2458 | 0 | 44 |
| B | bin11 | Enterocloster bolteae | GCF_002234575.2 | s__Enterocloster bolteae | 99.07 | 0.91 | 5,666,497 | 91 | 99,412 | 99.1 | 0.0 | 49.7 | 5122 | 0 | 65 |
| B | bin14 | Klebsiella quasipneumoniae | GCF_000751755.1 | s__Klebsiella quasipneumoniae | 96.40 | 0.93 | 4,645,257 | 357 | 20,589 | 65.2 | 0.0 | 58.8 | 4124 | 1 | 50 |
| B | bin7 | Citrobacter braakii | GCF_002075345.1 | s__Citrobacter braakii | 98.41 | 0.84 | 4,150,629 | 720 | 7,178 | 50.5 | 3.5 | 52.5 | 3421 | 2 | 43 |
| B | bin6 | Eggerthella lenta | GCF_000024265.1 | s__Eggerthella lenta | 98.15 | 0.97 | 1,786,663 | 631 | 3,009 | 54.1 | 0.1 | 64.3 | 1059 | 0 | 24 |
| C | bin3 | Acidaminococcus intestini | GCF_000425045.1 | s__Acidaminococcus intestini | 99.32 | 0.94 | 2,104,837 | 43 | 78,243 | 100.0 | 0.0 | 50.1 | 1950 | 2 | 49 |
| C | bin6 | Faecalimonas sp000209385 | GCA_902381685.1 | s__Faecalimonas sp000209385 | 99.08 | 0.85 | 2,323,081 | 73 | 59,966 | 98.7 | 0.0 | 38.9 | 2208 | 0 | 48 |
| C | bin27 | Blautia_A wexlerae | GCF_000484655.1 | s__Blautia_A wexlerae | 96.83 | 0.81 | 2,144,445 | 74 | 44,977 | 59.5 | 0.0 | 41.5 | 1930 | 0 | 16 |
| C | bin14 | Escherichia coli | GCF_003697165.2 | s__Escherichia coli | 99.04 | 0.95 | 4,608,175 | 225 | 38,266 | 99.5 | 1.3 | 50.9 | 4150 | 1 | 62 |
| C | bin13 | Peptostreptococcus anaerobius | GCF_000381525.1 | s__Peptostreptococcus anaerobius | 98.84 | 0.93 | 1,467,232 | 57 | 37,001 | 81.8 | 0.0 | 35.9 | 1277 | 2 | 3 |
| C | bin15 | Bacteroides uniformis | GCF_000154205.1 | s__Bacteroides uniformis | 96.21 | 0.81 | 3,523,373 | 331 | 16,433 | 93.0 | 3.3 | 47.2 | 2606 | 1 | 47 |
| C | bin9 | Peptostreptococcus stomatis | GCF_000147675.1 | s__Peptostreptococcus stomatis | 98.13 | 0.95 | 1,662,354 | 146 | 16,039 | 96.3 | 0.8 | 37.2 | 1399 | 2 | 23 |
| C | bin2 | Sutterella wadsworthensis_A | GCF_000297775.1 | s__Sutterella wadsworthensis_A | 98.88 | 0.95 | 2,041,725 | 364 | 7,328 | 87.9 | 2.6 | 62.9 | 1544 | 0 | 43 |
| C | bin20 | Erysipelatoclostridium ramosum | GCF_014131695.1 | s__Erysipelatoclostridium ramosum | 99.50 | 0.80 | 3,219,983 | 661 | 6,457 | 79.7 | 4.7 | 31.5 | 2735 | 0 | 37 |
| C | bin11 | Parvimonas sp000223315 | GCF_000223315.1 | s__Parvimonas sp000223315 | 97.67 | 0.95 | 1,223,673 | 361 | 3,892 | 65.4 | 0.7 | 28.2 | 960 | 0 | 27 |
| C | bin25 | Prevotella intermedia | GCF_001953955.1 | s__Prevotella intermedia | 96.44 | 0.98 | 1,112,041 | 397 | 2,880 | 54.6 | 1.4 | 44.3 | 640 | 0 | 6 |
| C | bin8 | Fusobacterium_C necrophorum | GCF_004006635.1 | s__Fusobacterium_C necrophorum | 96.08 | 0.90 | 1,087,070 | 413 | 2,689 | 59.4 | 0.0 | 36.0 | 747 | 0 | 5 |
| D | bin11 | Escherichia coli | GCF_003697165.2 | s__Escherichia coli | 98.33 | 0.95 | 4,545,689 | 65 | 156,505 | 99.5 | 0.6 | 50.7 | 4193 | 5 | 63 |
| D | bin23 | Acidaminococcus intestini | GCF_000425045.1 | s__Acidaminococcus intestini | 99.83 | 0.93 | 2,075,857 | 40 | 84,202 | 95.2 | 0.0 | 50.3 | 1936 | 2 | 48 |
| D | bin8 | Sellimonas intestinalis | GCF_001280875.1 | s__Sellimonas intestinalis | 99.47 | 0.91 | 2,914,926 | 107 | 52,951 | 95.9 | 2.0 | 45.7 | 2764 | 0 | 44 |
| D | bin18 | Clostridium_P perfringens | GCF_000013285.1 | s__Clostridium_P perfringens | 98.19 | 0.90 | 3,181,185 | 330 | 18,296 | 95.4 | 1.0 | 28.1 | 2732 | 5 | 68 |
| D | bin3 | Enterococcus faecalis | GCF_000392875.1 | s__Enterococcus faecalis | 98.84 | 0.97 | 2,527,558 | 248 | 14,196 | 98.7 | 1.3 | 37.8 | 2267 | 0 | 14 |
| D | bin20 | Holdemanella biformis | GCF_000156655.1 | s__Holdemanella biformis | 97.46 | 0.90 | 1,665,268 | 259 | 8,430 | 85.4 | 2.1 | 33.8 | 1548 | 0 | 31 |
| D | bin2 | Collinsella sp900541695 | GCA_008680965.1 | s__Collinsella aerofaciens_L | 94.92 | 0.90 | 1,845,426 | 365 | 6,237 | 83.5 | 1.8 | 60.2 | 1365 | 0 | 39 |
| D | bin19 | Phascolarctobacterium faecium | GCF_003269275.1 | s__Phascolarctobacterium faecium | 98.81 | 0.92 | 1,645,239 | 444 | 4,220 | 69.2 | 0.8 | 43.4 | 1291 | 0 | 41 |
| D | bin24 | Parabacteroides distasonis | GCF_000012845.1 | s__Parabacteroides distasonis | 96.69 | 0.95 | 2,218,373 | 786 | 2,908 | 56.7 | 1.7 | 45.2 | 1328 | 0 | 18 |
| E | bin27 | Escherichia coli | GCF_003697165.2 | s__Escherichia coli | 98.86 | 0.95 | 3,094,227 | 30 | 170,771 | 69.4 | 0.0 | 51.0 | 2839 | 6 | 53 |
| E | bin17 | Bacteroides thetaiotaomicron | GCF_000011065.1 | s__Bacteroides thetaiotaomicron | 98.16 | 0.85 | 5,691,866 | 142 | 77,011 | 97.9 | 2.1 | 43.0 | 4139 | 1 | 52 |
| E | bin7 | Acidaminococcus fermentans | GCF_000025305.1 | s__Acidaminococcus fermentans | 98.88 | 0.91 | 1,247,252 | 44 | 45,447 | 74.3 | 0.0 | 57.1 | 1191 | 1 | 29 |
| E | bin9 | Parvimonas micra | GCF_900637905.1 | s__Parvimonas micra | 97.57 | 0.90 | 1,678,607 | 74 | 42,216 | 98.5 | 1.2 | 28.4 | 1559 | 2 | 36 |
| E | bin13 | Collinsella sp900541675 | GCA_900759045.1 | s__Collinsella sp900759045 | 94.32 | 0.81 | 2,110,814 | 131 | 25,761 | 99.2 | 0.8 | 59.8 | 1797 | 0 | 53 |
| E | bin5 | Dorea_A longicatena_B | GCF_001404875.1 | s__Dorea_A longicatena_B | 97.19 | 0.82 | 2,854,571 | 222 | 23,519 | 95.2 | 4.5 | 41.9 | 2539 | 1 | 33 |
| E | bin18 | Eubacterium callanderi | GCF_900142645.1 | s__Eubacterium callanderi | 99.72 | 0.98 | 3,864,479 | 373 | 16,924 | 96.7 | 1.1 | 48.0 | 3375 | 1 | 37 |
| E | bin15 | Fusobacterium_B sp900541465 | GCA_900541465.1 | s__Fusobacterium_B sp900541465 | 97.22 | 0.77 | 2,177,661 | 316 | 9,878 | 96.6 | 1.1 | 30.2 | 1864 | 2 | 47 |
| E | bin10 | Phascolarctobacterium_A succinatutens | GCF_000188175.1 | s__Phascolarctobacterium_A succinatutens | 99.10 | 0.96 | 1,557,159 | 387 | 4,497 | 61.7 | 0.9 | 48.0 | 1202 | 0 | 28 |
| E | bin26 | Paraclostridium bifermentans | GCF_000452245.2 | s__Paraclostridium bifermentans_A | 96.37 | 0.90 | 3,045,640 | 804 | 4,446 | 93.1 | 2.5 | 28.3 | 2421 | 6 | 22 |
| F | bin17 | Escherichia coli | GCF_003697165.2 | s__Escherichia coli | 98.51 | 0.92 | 4,674,245 | 40 | 191,090 | 99.4 | 0.3 | 50.8 | 4330 | 4 | 63 |
| F | bin2 | Peptostreptococcus stomatis | GCF_000147675.1 | s__Peptostreptococcus stomatis | 97.93 | 0.87 | 1,183,289 | 12 | 161,199 | 58.0 | 0.0 | 37.3 | 1093 | 2 | 20 |
| F | bin4 | Megamonas funiformis | GCF_010669225.1 | s__Megamonas funiformis | 99.20 | 0.95 | 1,698,157 | 25 | 110,812 | 78.5 | 0.6 | 31.3 | 1638 | 3 | 45 |
| F | bin8 | Collinsella | GCA_018382125.1 | s__Collinsella sp018382125 | 93.85 | 0.85 | 2,108,869 | 103 | 32,051 | 98.4 | 1.6 | 59.9 | 1809 | 0 | 56 |
| F | bin1 | Bifidobacterium adolescentis | GCF_000010425.1 | s__Bifidobacterium adolescentis | 98.07 | 0.85 | 2,049,800 | 166 | 16,942 | 98.2 | 0.6 | 59.6 | 1641 | 3 | 52 |
| F | bin15 | Bacteroides uniformis | GCF_000154205.1 | s__Bacteroides uniformis | 99.24 | 0.88 | 3,229,526 | 294 | 16,388 | 79.6 | 3.9 | 48.0 | 2358 | 0 | 35 |
| F | bin21 | Streptococcus oralis_W | GCF_000257845.1 | s__Streptococcus oralis_W | 97.58 | 0.97 | 1,745,149 | 170 | 15,733 | 96.6 | 0.9 | 41.5 | 1547 | 1 | 24 |
| F | bin14 | Fusobacterium_A ulcerans_A | GCF_900683735.1 | s__Fusobacterium_A ulcerans_A | 99.98 | 0.98 | 3,481,470 | 370 | 14,571 | 98.9 | 4.5 | 29.7 | 3078 | 6 | 53 |
| F | bin3 | Gemella morbillorum | GCF_900476045.1 | s__Gemella morbillorum | 98.33 | 0.92 | 1,650,305 | 242 | 9,498 | 92.3 | 1.2 | 30.5 | 1430 | 3 | 34 |
| F | bin24 | Collinsella tanakaei | GCF_000225705.1 | s__Collinsella tanakaei | 95.50 | 0.90 | 1,929,567 | 365 | 6,557 | 84.6 | 0.8 | 61.0 | 1432 | 0 | 38 |
| G | bin11 | Clostridium_X cadaveris | GCF_000424205.1 | s__Clostridium_X cadaveris | 99.46 | 0.92 | 3,451,105 | 93 | 61,079 | 93.4 | 2.5 | 31.1 | 3279 | 5 | 57 |
| G | bin1 | Bacteroides thetaiotaomicron | GCF_000011065.1 | s__Bacteroides thetaiotaomicron | 97.96 | 0.81 | 6,268,823 | 229 | 53,377 | 81.2 | 2.6 | 42.8 | 4615 | 0 | 51 |
| G | bin3 | Peptostreptococcus stomatis | GCF_000147675.1 | s__Peptostreptococcus stomatis | 97.55 | 0.84 | 1,155,416 | 27 | 51,084 | 51.8 | 0.0 | 36.0 | 1070 | 0 | 1 |
| G | bin14 | Anaerostipes hadrus | GCF_000332875.2 | s__Anaerostipes hadrus | 98.18 | 0.82 | 2,639,109 | 104 | 45,538 | 96.0 | 2.7 | 37.2 | 2487 | 0 | 56 |
| G | bin16 | Paraclostridium bifermentans | GCF_006802875.1 | s__Paraclostridium bifermentans | 97.25 | 0.89 | 3,539,229 | 215 | 28,189 | 82.8 | 0.0 | 28.0 | 3374 | 6 | 33 |
| G | bin20 | Bacteroides pyogenes | GCF_000428105.1 | s__Bacteroides pyogenes | 99.17 | 0.95 | 2,516,707 | 268 | 14,312 | 87.0 | 2.1 | 47.0 | 1794 | 0 | 41 |
| G | bin12 | Prevotella denticola | GCF_900454835.1 | s__Prevotella denticola | 97.88 | 0.93 | 2,499,482 | 270 | 13,574 | 92.1 | 0.9 | 51.0 | 1749 | 2 | 34 |
| G | bin23 | Collinsella | GCA_905214435.1 | s__Collinsella sp905214435 | 94.63 | 0.82 | 2,048,708 | 232 | 12,387 | 96.8 | 2.1 | 60.0 | 1658 | 0 | 47 |
| G | bin22 | Enterococcus_B lactis | GCF_015751045.1 | s__Enterococcus_B lactis | 98.08 | 0.93 | 2,476,146 | 354 | 9,767 | 94.3 | 4.9 | 38.5 | 2113 | 2 | 31 |
| G | bin18 | Clostridium_Q symbiosum | GCF_000466485.1 | s__Clostridium_Q symbiosum | 98.75 | 0.88 | 3,772,118 | 950 | 4,534 | 77.6 | 3.7 | 48.3 | 2811 | 0 | 25 |
| G | bin5 | Oribacterium sp013394775 | GCF_013394775.1 | s__Oribacterium sp013394775 | 98.69 | 0.96 | 1,765,507 | 465 | 4,308 | 76.0 | 0.2 | 56.4 | 1187 | 0 | 33 |
| G | bin2 | Eubacterium_B infirmum | GCA_900450455.1 | s__Eubacterium_B infirmum | 97.77 | 0.97 | 1,120,653 | 311 | 4,299 | 65.6 | 0.2 | 41.6 | 811 | 1 | 9 |
| G | bin15 | Erysipelatoclostridium ramosum | GCF_014131695.1 | s__Erysipelatoclostridium ramosum | 99.71 | 0.98 | 1,341,211 | 566 | 2,359 | 51.6 | 0.5 | 31.8 | 883 | 0 | 6 |
| H | bin19 | Ruminococcus_B gnavus | GCF_008121495.1 | s__Ruminococcus_B gnavus | 98.91 | 0.94 | 1,187,910 | 21 | 102,915 | 55.2 | 0.0 | 43.6 | 1133 | 0 | 11 |
| H | bin8 | Escherichia coli | GCF_003697165.2 | s__Escherichia coli | 98.76 | 0.95 | 4,625,577 | 110 | 79,830 | 99.6 | 0.1 | 50.8 | 4241 | 4 | 65 |
| H | bin17 | Faecalimonas phoceensis | GCF_900104635.1 | s__Faecalimonas phoceensis | 99.15 | 0.93 | 2,377,548 | 62 | 67,675 | 89.0 | 1.9 | 40.6 | 2182 | 0 | 48 |
| H | bin5 | Lactococcus formosensis | GCF_018403745.1 | s__Lactococcus formosensis | 97.98 | 0.91 | 1,260,898 | 32 | 61,636 | 53.6 | 0.0 | 37.6 | 1233 | 0 | 12 |
| H | bin3 | Anaerotignum lactatifermentans | GCF_900142265.1 | s__Anaerotignum lactatifermentans | 97.83 | 0.88 | 2,620,801 | 116 | 36,871 | 87.4 | 0.3 | 45.2 | 2548 | 2 | 41 |
| H | bin21 | Clostridium_Q symbiosum | GCF_000466485.1 | s__Clostridium_Q symbiosum | 98.97 | 0.85 | 4,515,195 | 255 | 25,368 | 98.7 | 0.6 | 48.0 | 3954 | 0 | 55 |
| H | bin10 | Megasphaera elsdenii | GCF_003010495.1 | s__Megasphaera elsdenii | 98.25 | 0.95 | 2,030,402 | 189 | 16,291 | 97.6 | 0.3 | 54.0 | 1753 | 0 | 45 |
| H | bin18 | Flavonifractor plautii | GCF_000239295.1 | s__Flavonifractor plautii | 98.21 | 0.88 | 3,096,026 | 630 | 5,972 | 91.6 | 2.0 | 62.0 | 2686 | 0 | 31 |
| H | bin1 | Streptococcus pasteurianus | GCA_900478025.1 | s__Streptococcus pasteurianus | 99.22 | 0.96 | 1,459,666 | 348 | 5,031 | 80.0 | 1.9 | 37.7 | 1192 | 0 | 24 |
| H | bin13 | Collinsella aerofaciens_M | GCF_019041915.1 | s__Collinsella sp019041915 | 94.35 | 0.90 | 1,761,505 | 425 | 4,658 | 86.5 | 2.2 | 60.1 | 1233 | 1 | 31 |
| H | bin22 | Bifidobacterium adolescentis | GCF_000010425.1 | s__Bifidobacterium adolescentis | 97.86 | 0.95 | 1,372,382 | 416 | 3,530 | 75.5 | 2.3 | 60.1 | 800 | 0 | 40 |
| I | bin20 | Clostridium_AQ innocuum | GCF_012317185.1 | s__Clostridium_AQ innocuum | 97.43 | 0.95 | 1,323,596 | 20 | 102,481 | 56.1 | 0.0 | 45.4 | 1318 | 1 | 15 |
| I | bin11 | Bacteroides thetaiotaomicron | GCF_000011065.1 | s__Bacteroides thetaiotaomicron | 99.02 | 0.90 | 5,596,241 | 93 | 92,582 | 72.1 | 4.5 | 42.9 | 4053 | 4 | 44 |
| I | bin5 | Faecalimonas phoceensis | GCF_900104635.1 | s__Faecalimonas phoceensis | 98.73 | 0.82 | 3,068,405 | 123 | 64,174 | 99.4 | 1.3 | 40.0 | 2882 | 0 | 56 |
| I | bin14 | Blautia coccoides | GCF_900461125.1 | s__Blautia coccoides | 99.59 | 0.93 | 5,567,591 | 178 | 52,406 | 96.7 | 2.8 | 46.0 | 4873 | 1 | 58 |
| I | bin4 | Clostridium_Q symbiosum | GCF_000466485.1 | s__Clostridium_Q symbiosum | 99.06 | 0.82 | 4,826,597 | 194 | 35,933 | 93.7 | 0.7 | 48.1 | 4226 | 0 | 54 |
| I | bin16 | Collinsella | N/A | N/A | N/A | N/A | 2,095,200 | 114 | 25,992 | 98.4 | 0.8 | 60.1 | 1788 | 0 | 54 |
| I | bin17 | Escherichia coli | GCF_003697165.2 | s__Escherichia coli | 98.73 | 0.94 | 4,534,470 | 299 | 21,944 | 98.5 | 2.8 | 50.9 | 3981 | 4 | 65 |
| I | bin25 | Bacteroides uniformis | GCF_000154205.1 | s__Bacteroides uniformis | 98.30 | 0.88 | 3,982,151 | 361 | 17,196 | 94.1 | 2.5 | 46.7 | 2881 | 0 | 42 |
| I | bin18 | Ruminococcus_B gnavus | GCF_008121495.1 | s__Ruminococcus_B gnavus | 97.74 | 0.84 | 3,020,630 | 297 | 17,084 | 98.3 | 3.3 | 43.2 | 2749 | 0 | 56 |
| I | bin22 | Erysipelatoclostridium ramosum | GCF_014131695.1 | s__Erysipelatoclostridium ramosum | 99.72 | 0.80 | 3,969,189 | 686 | 8,555 | 94.3 | 1.9 | 32.0 | 3439 | 0 | 39 |
| I | bin2 | Flavonifractor plautii | GCF_000239295.1 | s__Flavonifractor plautii | 98.33 | 0.87 | 3,144,419 | 510 | 8,139 | 94.5 | 0.8 | 62.4 | 2845 | 0 | 35 |
| I | bin10 | Paraeggerthella hongkongensis_A | GCF_003340345.1 | s__Paraeggerthella hongkongensis_A | 98.41 | 0.92 | 2,448,141 | 434 | 7,573 | 92.9 | 1.2 | 61.6 | 1850 | 0 | 47 |
| I | bin26 | Enterocloster bolteae | GCF_002234575.2 | s__Enterocloster bolteae | 97.30 | 0.90 | 4,608,478 | 1,077 | 5,287 | 77.0 | 1.8 | 49.9 | 3408 | 0 | 41 |
| J | bin4 | Streptococcus anginosus_C | GCF_001697145.1 | s__Streptococcus anginosus_C | 97.25 | 0.88 | 1,191,555 | 16 | 192,769 | 52.6 | 0.0 | 38.7 | 1163 | 0 | 13 |
| J | bin7 | Anaerotignum sp001304995 | GCA_001304995.1 | s__Anaerotignum sp001304995 | 98.95 | 0.86 | 1,740,182 | 25 | 129,964 | 68.8 | 0.0 | 46.7 | 1729 | 1 | 49 |
| J | bin23 | Bacteroides fragilis | GCF_000025985.1 | s__Bacteroides fragilis | 99.13 | 0.91 | 4,846,791 | 151 | 65,214 | 97.4 | 1.3 | 43.4 | 3777 | 2 | 55 |
| J | bin11 | Clostridium_Q symbiosum | GCF_000466485.1 | s__Clostridium_Q symbiosum | 98.86 | 0.82 | 4,488,143 | 301 | 21,572 | 97.5 | 0.0 | 48.0 | 3962 | 0 | 38 |
| J | bin2 | Collinsella | N/A | N/A | N/A | N/A | 2,099,670 | 155 | 19,982 | 99.7 | 0.8 | 60.1 | 1778 | 0 | 50 |
| J | bin9 | Sutterella wadsworthensis_A | GCF_000297775.1 | s__Sutterella wadsworthensis_A | 98.83 | 0.96 | 2,239,502 | 209 | 15,444 | 96.6 | 0.6 | 62.8 | 1857 | 1 | 60 |
| J | bin20 | Erysipelatoclostridium ramosum | GCF_014131695.1 | s__Erysipelatoclostridium ramosum | 99.75 | 0.98 | 2,947,183 | 311 | 14,202 | 93.9 | 0.9 | 31.4 | 2623 | 0 | 36 |
| J | bin22 | Sellimonas intestinalis | GCF_001280875.1 | s__Sellimonas intestinalis | 99.53 | 0.94 | 2,485,425 | 420 | 7,893 | 87.2 | 3.1 | 46.7 | 2044 | 0 | 28 |
| J | bin5 | Bifidobacterium pseudocatenulatum | GCF_001025215.1 | s__Bifidobacterium pseudocatenulatum | 97.71 | 0.93 | 1,613,717 | 462 | 3,849 | 76.5 | 4.7 | 56.5 | 1000 | 1 | 45 |
| J | bin10 | Alistipes onderdonkii | GCF_000374505.1 | s__Alistipes onderdonkii | 97.24 | 0.96 | 1,618,444 | 579 | 2,967 | 59.3 | 1.5 | 59.8 | 883 | 0 | 21 |
| J | bin14 | Eubacterium callanderi | GCF_900142645.1 | s__Eubacterium callanderi | 98.45 | 0.95 | 1,813,282 | 676 | 2,792 | 51.4 | 0.4 | 47.9 | 1240 | 2 | 23 |
| K | bin9 | Escherichia coli | GCF_011881725.1 | s__Escherichia coli_E | 92.57 | 0.79 | 2,805,887 | 28 | 205,053 | 58.6 | 0.0 | 51.0 | 2535 | 1 | 47 |
| K | bin22 | Parabacteroides merdae | GCF_000154105.1 | s__Parabacteroides merdae | 99.43 | 0.93 | 3,163,156 | 69 | 92,492 | 73.9 | 1.2 | 46.1 | 2399 | 0 | 48 |
| K | bin15 | Ruminococcus_B gnavus | GCF_008121495.1 | s__Ruminococcus_B gnavus | 99.30 | 0.93 | 2,535,505 | 70 | 65,491 | 92.6 | 0.0 | 43.6 | 2375 | 1 | 45 |
| K | bin29 | Blautia_A wexlerae | GCF_000484655.1 | s__Blautia_A wexlerae | 96.67 | 0.74 | 1,820,386 | 81 | 36,343 | 54.4 | 0.0 | 41.2 | 1652 | 0 | 21 |
| K | bin8 | Coprococcus_A sp900548825 | GCF_018785175.1 | s__Coprococcus_A sp900548825 | 98.04 | 0.90 | 2,779,750 | 220 | 20,711 | 96.6 | 2.2 | 43.6 | 2427 | 0 | 54 |
| K | bin10 | Collinsella | N/A | N/A | N/A | N/A | 2,152,646 | 169 | 18,466 | 96.3 | 2.0 | 60.0 | 1807 | 2 | 53 |
| K | bin23 | Phascolarctobacterium_A succinatutens | GCF_000188175.1 | s__Phascolarctobacterium_A succinatutens | 95.07 | 0.86 | 1,993,151 | 246 | 10,537 | 95.7 | 0.9 | 47.8 | 1665 | 3 | 37 |
| K | bin17 | Clostridium_Q symbiosum | GCF_000466485.1 | s__Clostridium_Q symbiosum | 98.84 | 0.92 | 3,890,215 | 624 | 8,437 | 84.1 | 2.8 | 48.3 | 3106 | 0 | 42 |
| K | bin5 | Bifidobacterium longum | GCF_000196555.1 | s__Bifidobacterium longum | 98.99 | 0.94 | 1,214,466 | 395 | 3,319 | 63.1 | 0.8 | 60.3 | 677 | 0 | 26 |
| K | bin12 | Megamonas funiformis | GCF_010669225.1 | s__Megamonas funiformis | 97.91 | 0.91 | 1,352,276 | 441 | 3,269 | 56.0 | 0.0 | 31.9 | 965 | 1 | 26 |
| K | bin6 | Parabacteroides distasonis | GCF_000012845.1 | s__Parabacteroides distasonis | 98.28 | 0.96 | 1,853,076 | 631 | 3,111 | 51.2 | 0.0 | 45.8 | 1012 | 0 | 14 |

^a^ Taxonomic classification was performed using the Genome Taxonomy Database toolkit (GTDB-Tk, v2.0.0) against GTDB release 207, with option --full_tree.

^b^ Output as reported by GTDB-Tk’s classify_wf workflow (ANI: average nucleotide identity; AF: alignment fraction). N/A indicates that the MAGs failed to match a representative genome within the species’ ANI radius.

Columns indicate the following: closest_placement_reference, indicates the accession number of the reference genome when a genome is placed on a terminal branch; closest_placement_taxonomy, indicates the GTDB taxonomy of the reference genome; closest_placement_ani, indicates the ANI between the query and above reference genome.; closest_placement_af, indicates the alignment fraction (AF) between the query and the above reference genome.

^c^ Estimation of completeness and contamination of MAGs was performed using CheckM (v1.13) .

^d^ Annotation was performed using the DDBJ Fast Annotation and Submission Tool (DFAST, v1.2.15).

**Supplementary Table S6.** Summary of MarkerMAG analysis for linking MAGs to 16S rRNA gene sequence and ASVs.

| **Patient identifier** | **MAG**  **identifier** | **No.**  **of links** | **Linked**  **ASV** | **Taxonomic classification of MAG**  **(GTDB, release 207)** | **Taxonomic classification of MarkerMAG-linked ASV**  **(Silva, release 138)** |
| --- | --- | --- | --- | --- | --- |
|  |  |  |  |  |  |
| A | bin2 | 23 | asv17 | Bacilli;Lactobacillales;Streptococcaceae;Streptococcus;Streptococcus salivarius | Bacilli;Lactobacillales;Streptococcaceae;Streptococcus |
| A | bin25 | 270 | asv301 | Negativicutes;Acidaminococcales;Acidaminococcaceae;Acidaminococcus;Acidaminococcus sp000437815 | Negativicutes;Acidaminococcales;Acidaminococcaceae;Acidaminococcus |
| A | bin4 | 57 | asv76 | Bacilli;Lactobacillales;Streptococcaceae;Streptococcus;Streptococcus anginosus | Bacilli;Lactobacillales;Streptococcaceae;Streptococcus |
| B | bin10 | 50 | asv265 | Bacilli;Lactobacillales;Enterococcaceae;Enterococcus;Enterococcus faecalis | Bacilli;Lactobacillales;Enterococcaceae;Enterococcus |
| B | bin11 | 28 | asv165 | Clostridia;Lachnospirales;Lachnospiraceae;Enterocloster;Enterocloster bolteae | Clostridia;Lachnospirales;Lachnospiraceae;Lachnoclostridium |
| B | bin14 | 19 | asv57 | Gammaproteobacteria;Enterobacterales;Enterobacteriaceae;Klebsiella;Klebsiella quasipneumoniae | Gammaproteobacteria;Enterobacterales;Enterobacteriaceae;Escherichia-Shigella |
| B | bin7 | 14 | asv1173 | Gammaproteobacteria;Enterobacterales;Enterobacteriaceae;Citrobacter;Citrobacter braakii | Gammaproteobacteria;Enterobacterales;Enterobacteriaceae;Citrobacter |
| C | bin11 | 10 | asv52 | Clostridia;Tissierellales;Peptoniphilaceae;Parvimonas;Parvimonas sp000223315 | Clostridia;PeptostreptococcalesTissierellales;Family XI;Parvimonas |
| C | bin13 | 91 | asv77 | Clostridia;Peptostreptococcales;Peptostreptococcaceae;Peptostreptococcus;Peptostreptococcus anaerobius | Clostridia;PeptostreptococcalesTissierellales;Peptostreptococcaceae;Peptostreptococcus |
| C | bin14 | 60 | asv57 | Gammaproteobacteria;Enterobacterales;Enterobacteriaceae;Escherichia;Escherichia coli | Gammaproteobacteria;Enterobacterales;Enterobacteriaceae;Escherichia-Shigella |
| C | bin20 | 17 | asv74 | Bacilli;Erysipelotrichales;Coprobacillaceae;Erysipelatoclostridium;Erysipelatoclostridium ramosum | Bacilli;Erysipelotrichales;Erysipelatoclostridiaceae;Erysipelatoclostridium |
| C | bin3 | 50 | asv176 | Negativicutes;Acidaminococcales;Acidaminococcaceae;Acidaminococcus;Acidaminococcus intestini | Negativicutes;Acidaminococcales;Acidaminococcaceae;Acidaminococcus |
| C | bin6 | 14 | asv118 | Clostridia;Lachnospirales;Lachnospiraceae;Faecalimonas;Faecalimonas sp000209385 | Clostridia;Lachnospirales;Lachnospiraceae;Lachnoclostridium |
| C | bin8 | 11 | asv14 | Fusobacteriia;Fusobacteriales;Fusobacteriaceae;Fusobacterium_C;Fusobacterium_C necrophorum | Fusobacteriia;Fusobacteriales;Fusobacteriaceae;Fusobacterium |
| C | bin9 | 11 | asv44 | Clostridia;Peptostreptococcales;Peptostreptococcaceae;Peptostreptococcus;Peptostreptococcus stomatis | Clostridia;PeptostreptococcalesTissierellales;Peptostreptococcaceae;Peptostreptococcus |
| D | bin11 | 139 | asv57 | Gammaproteobacteria;Enterobacterales;Enterobacteriaceae;Escherichia;Escherichia coli | Gammaproteobacteria;Enterobacterales;Enterobacteriaceae;Escherichia-Shigella |
| D | bin18 | 175 | asv172 | Clostridia;Clostridiales;Clostridiaceae;Clostridium_P;Clostridium_P perfringens | Clostridia;Clostridiales;Clostridiaceae;Clostridium sensu stricto 1 |
| D | bin19 | 19 | asv229 | Negativicutes;Acidaminococcales;Acidaminococcaceae;Phascolarctobacterium;Phascolarctobacterium faecium | Negativicutes;Acidaminococcales;Acidaminococcaceae;Phascolarctobacterium |
| D | bin20 | 12 | asv225 | Bacilli;Erysipelotrichales;Erysipelotrichaceae;Holdemanella;Holdemanella biformis | Bacilli;Erysipelotrichales;Erysipelotrichaceae;Holdemanella |
| D | bin23 | 756 | asv176 | Negativicutes;Acidaminococcales;Acidaminococcaceae;Acidaminococcus;Acidaminococcus intestini | Negativicutes;Acidaminococcales;Acidaminococcaceae;Acidaminococcus |
| D | bin3 | 30 | asv265 | Firmicutes;Bacilli;Lactobacillales;Enterococcaceae;Enterococcus;Enterococcus faecalis | Bacilli;Lactobacillales;Enterococcaceae;Enterococcus |
| D | bin8 | 11 | asv240 | Firmicutes_A;Clostridia;Lachnospirales;Lachnospiraceae;Sellimonas;Sellimonas intestinalis | Clostridia;Lachnospirales;Lachnospiraceae;Sellimonas |
| E | bin13 | 33 | asv1 | Coriobacteriia;Coriobacteriales;Coriobacteriaceae;Collinsella;Collinsella sp900541675 | Coriobacteriia;Coriobacteriales;Coriobacteriaceae;Collinsella |
| E | bin15 | 23 | asv28 | Fusobacteriia;Fusobacteriales;Fusobacteriaceae;Fusobacterium_B;Fusobacterium_B sp900541465 | Fusobacteriia;Fusobacteriales;Fusobacteriaceae;Fusobacterium |
| E | bin18 | 53 | asv313 | Clostridia;Eubacteriales;Eubacteriaceae;Eubacterium;Eubacterium callanderi | Clostridia;Eubacteriales;Eubacteriaceae;Eubacterium |
| E | bin26 | 160 | asv1563 | Clostridia;Peptostreptococcales;Peptostreptococcaceae;Paraclostridium;Paraclostridium bifermentans | Clostridia;PeptostreptococcalesTissierellales;Peptostreptococcaceae;Paraclostridium |
| E | bin27 | 237 | asv57 | Gammaproteobacteria;Enterobacterales;Enterobacteriaceae;Escherichia;Escherichia coli | Gammaproteobacteria;Enterobacterales;Enterobacteriaceae;Escherichia-Shigella |
| E | bin9 | 46 | asv21 | Clostridia;Tissierellales;Peptoniphilaceae;Parvimonas;Parvimonas micra | Clostridia;PeptostreptococcalesTissierellales;Family XI;Parvimonas |
| F | bin14 | 86 | asv175 | Fusobacteriia;Fusobacteriales;Fusobacteriaceae;Fusobacterium_A;Fusobacterium_A ulcerans_A | Fusobacteriia;Fusobacteriales;Fusobacteriaceae;Fusobacterium |
| F | bin17 | 104 | asv57 | Gammaproteobacteria;Enterobacterales;Enterobacteriaceae;Escherichia;Escherichia coli | Gammaproteobacteria;Enterobacterales;Enterobacteriaceae;Escherichia-Shigella |
| F | bin2 | 88 | asv44 | Clostridia;Peptostreptococcales;Peptostreptococcaceae;Peptostreptococcus;Peptostreptococcus stomatis | Clostridia;PeptostreptococcalesTissierellales;Peptostreptococcaceae;Peptostreptococcus |
| F | bin3 | 36 | asv78 | Bacilli;Staphylococcales;Gemellaceae;Gemella;Gemella morbillorum | Bacilli;Staphylococcales;Gemellaceae;Gemella |
| F | bin4 | 88 | asv33 | Negativicutes;Selenomonadales;Selenomonadaceae;Megamonas;Megamonas funiformis | Negativicutes;Veillonellales-Selenomonadales;Selenomonadaceae;Megamonas |
| F | bin8 | 14 | asv1 | Coriobacteriia;Coriobacteriales;Coriobacteriaceae;Collinsella; | Coriobacteriia;Coriobacteriales;Coriobacteriaceae;Collinsella |
| G | bin11 | 150 | asv660 | Clostridia;Clostridiales;Clostridiaceae;Clostridium_X;Clostridium_X cadaveris | Clostridia;Clostridiales;Clostridiaceae;Clostridium sensu stricto 2 |
| G | bin14 | 19 | asv46 | Clostridia;Lachnospirales;Lachnospiraceae;Anaerostipes;Anaerostipes hadrus | Clostridia;Lachnospirales;Lachnospiraceae;Anaerostipes |
| G | bin16 | 134 | asv1563 | Clostridia;Peptostreptococcales;Peptostreptococcaceae;Paraclostridium;Paraclostridium bifermentans | Clostridia;Peptostreptococcales-Tissierellales;Peptostreptococcaceae;Paraclostridium |
| G | bin22 | 26 | asv265 | Bacilli;Lactobacillales;Enterococcaceae;Enterococcus_B;Enterococcus_B lactis | Bacilli;Lactobacillales;Enterococcaceae;Enterococcus |
| G | bin23 | 10 | asv1 | Coriobacteriia;Coriobacteriales;Coriobacteriaceae;Collinsella; | Coriobacteriia;Coriobacteriales;Coriobacteriaceae;Collinsella |
| H | bin1 | 18 | asv297 | Lactobacillales;Streptococcaceae;Streptococcus;Streptococcus pasteurianus | Bacilli;Lactobacillales;Streptococcaceae;Streptococcus |
| H | bin10 | 16 | asv70 | Negativicutes;Veillonellales;Megasphaeraceae;Megasphaera;Megasphaera elsdenii | Negativicutes;Veillonellales-Selenomonadales;Veillonellaceae;Megasphaera |
| H | bin21 | 40 | asv26 | Clostridia;Lachnospirales;Lachnospiraceae;Clostridium_Q;Clostridium_Q symbiosum | Clostridia;Lachnospirales;Lachnospiraceae;[Ruminococcus] torques group |
| H | bin8 | 57 | asv57 | Gammaproteobacteria;Enterobacterales;Enterobacteriaceae;Escherichia;Escherichia coli | Gammaproteobacteria;Enterobacterales;Enterobacteriaceae;Escherichia-Shigella |
| I | bin10 | 9 | asv501 | Coriobacteriia;Coriobacteriales;Eggerthellaceae;Paraeggerthella;Paraeggerthella hongkongensis_A | Coriobacteriia;Coriobacteriales;Eggerthellaceae;Paraeggerthella |
| I | bin11 | 9 | asv39 | Bacteroidia;Bacteroidales;Bacteroidaceae;Bacteroides;Bacteroides thetaiotaomicron | Bacteroidia;Bacteroidales;Bacteroidaceae;Bacteroides |
| I | bin14 | 28 | asv398 | Clostridia;Lachnospirales;Lachnospiraceae;Blautia;Blautia coccoides | Clostridia;Lachnospirales;Lachnospiraceae;Blautia |
| I | bin16 | 18 | asv1 | Coriobacteriia;Coriobacteriales;Coriobacteriaceae;Collinsella | Coriobacteriia;Coriobacteriales;Coriobacteriaceae;Collinsella |
| I | bin17 | 110 | asv57 | Gammaproteobacteria;Enterobacterales;Enterobacteriaceae;Escherichia;Escherichia coli | Gammaproteobacteria;Enterobacterales;Enterobacteriaceae;Escherichia-Shigella |
| I | bin18 | 21 | asv8 | Clostridia;Lachnospirales;Lachnospiraceae;Ruminococcus_B;Ruminococcus_B gnavus | Clostridia;Lachnospirales;Lachnospiraceae;[Ruminococcus] gnavus group |
| I | bin22 | 44 | asv74 | Bacilli;Erysipelotrichales;Coprobacillaceae;Erysipelatoclostridium;Erysipelatoclostridium ramosum | Bacilli;Erysipelotrichales;Erysipelatoclostridiaceae;Erysipelatoclostridium |
| I | bin4 | 40 | asv26 | Clostridia;Lachnospirales;Lachnospiraceae;Clostridium_Q;Clostridium_Q symbiosum | Clostridia;Lachnospirales;Lachnospiraceae;[Ruminococcus] torques group |
| I | bin5 | 30 | asv413 | Clostridia;Lachnospirales;Lachnospiraceae;Faecalimonas;Faecalimonas phoceensis | Clostridia;Lachnospirales;Lachnospiraceae;[Ruminococcus] torques group |
| J | bin11 | 12 | asv19 | Clostridia;Lachnospirales;Lachnospiraceae;Clostridium_Q;Clostridium_Q symbiosum | Bacteroidia;Bacteroidales;Bacteroidaceae;Bacteroides |
| J | bin2 | 16 | asv1 | Coriobacteriia;Coriobacteriales;Coriobacteriaceae;Collinsella | Coriobacteriia;Coriobacteriales;Coriobacteriaceae;Collinsella |
| J | bin20 | 18 | asv74 | Bacilli;Erysipelotrichales;Coprobacillaceae;Erysipelatoclostridium;Erysipelatoclostridium ramosum | Bacilli;Erysipelotrichales;Erysipelatoclostridiaceae;Erysipelatoclostridium |
| J | bin9 | 81 | asv27 | Gammaproteobacteria;Burkholderiales;Burkholderiaceae;Sutterella;Sutterella wadsworthensis_A | Gammaproteobacteria;Burkholderiales;Sutterellaceae;Sutterella |
| K | bin22 | 16 | asv18 | Bacteroidota;Bacteroidia;Bacteroidales;Tannerellaceae;Parabacteroides;Parabacteroides merdae | Bacteroidia;Bacteroidales;Tannerellaceae;Parabacteroides |
| K | bin29 | 14 | asv96 | Clostridia;Lachnospirales;Lachnospiraceae;Blautia_A;Blautia_A wexlerae | Bacteroidia;Bacteroidales;Bacteroidaceae;Bacteroides |
| K | bin9 | 202 | asv57 | Gammaproteobacteria;Enterobacterales;Enterobacteriaceae;Escherichia;Escherichia coli | Gammaproteobacteria;Enterobacterales;Enterobacteriaceae;Escherichia-Shigella |

**Supplementary Table S7.** Overview of putative fadA proteins encoded in MAG bin14 from patient F.

| **Predicted protein sequence in MAG** | **E-value match**  **in Pfam database ^a^** | **Score match**  **in Pfam database ^a^** | **Best match in NCBI’s non-redundant protein database ^b^** |
| --- | --- | --- | --- |
|  |  |  |  |
| MKKLLVAGTILLSATAFSTGVTAEFESRFNTLEQEYKMLMQKEDER  YNSEKQIAETAKATLAKQRELYNQISTKSAKLGQIKDVKFYKEQYG  ELAKKYQDALRELEGQMKEQESIINRFQQLQAVKEGK | 1.70×10^-29^ | 99.7 | adhesion protein FadA, *Fusobacterium ulcerans*  Accession number: WP_130890606.1  Identities: 129/129 (100%)  Positives: 129/129 (100%) |
|  |  |  |  |
| MKIKTTMLLGAALLLVSSVSLAAPAAGVDSRFSQLEAELKMLEQKE  NERFKEEEQIAKSAQNNLNALTNLRNKCGERINYMTSMEGRSIYSN  EMKNLLKQYQGFLTEIDKQSKVEERKIFEFNQLKSLRAE | 1.20×10^-27^ | 93.8 | adhesion protein FadA, *Fusobacterium ulcerans*  Accession number: WP_130890605.1  Identities: 131/131 (100%)  Positives: 131/131 (100%) |
|  |  |  |  |
| MKKILVGCFLAVSAISYSATDVMSTFEQLELNLQQLEAEERAMYNQ  RKAEAEEAEKTLAAQRKMYAEISEKEKRILSVKDNKFYKTQYQELA  KKYSEAKKELEKDMKRQEEIISIFEAIR | 2.70×10^-20^ | 70.2 | adhesion protein FadA, *Fusobacterium ulcerans*  Accession number: WP_130892092.1  Identities: 120/120 (100%)  Positives: 120/120 (100%) |
|  |  |  |  |
| MKKILIGCILAVSAVSYSATDVMSTFEQLELNLQQLEAEERAMYNQ  RKAEAEEAEKTLAAQRKMYAEISEKEKRIISVKDNKFYKDQYQELA  RKYGEAKKELEIDMGKHEEIIKMFEVIK | 3.20×10^-18^ | 63.6 | adhesin, *Fusobacterium ulcerans*  Accession number: WP_130889439.1  Identities: 119/120 (99%)  Positives: 119/120 (99%) |
|  |  |  |  |
| MRKVLLGCFLFTASITIFAETNVLSTLEQLELNFQQLEAEEKAMYE  KRKSEAEEAQRTLAQQREMYQQIITQEKRIADVKGNRYYKDQYNQL  AKKYSDAKKVLEEDMKKQEEIINLFEMIK | 3.30×10^-18^ | 63.5 | adhesin, *Fusobacterium ulcerans*  Accession number: WP_130889290.1  Identities: 121/121(100%)  Positives: 121/121(100%) |
|  |  |  |  |
| MKKILLGCLLVVSVTAFAATDVMVTLEQLEQNFQQLEAEERAMYDQ  RKAEAETAEKVLAEQKATYQQIIAQERRIADVKEFRYYKGQYNQLA  KKYSDAKRTLEDEMKKQEEIIYMFEIMK | 1.90×10^-17^ | 61.1 | adhesin, *Fusobacterium ulcerans*  Accession number: WP_130889935.1  Identities: 120/120 (100%)  Positives: 120/120 (100%) |
|  |  |  |  |
| MKKILIGCVLVISTVSYSAIEAVSTFEQLELTFQQLEAEEAAMYNQ  RKTEAEEAEKVLVSLRAKYQKILETEKYIIEVEQYRYYQEDYKELL  KKCRTMKGELEAEIAAKEEIIDIYRAIM | 1.50×10^-13^ | 48.6 | adhesin, *Fusobacterium ulcerans*  Accession number: WP_130890415.1  Identities: 120/120 (100%)  Positives: 120/120 (100%) |

## Supplementary Figures

**Supplementary Figure S1.** Overview of samples collected in this study. The number of samples collected at each location and patient are indicated. Negative and positive distances on the *x*-axis indicate samples toward the oral and anal side of the colon with respect to the tumor, respectively.

**Supplementary Figure S2.** Taxonomic composition of mucosa-associated microbiota across samples. Stacked bar charts show the relative abundance of the most prevalent phyla and genera. Fill colors reflect phyla, and genera within a phylum are shown with varying hues. Distances between the sampling location and tumor are shown on the *x*-axis, with negative and positive distances indicating samples toward the oral and anal side of the colon with respect to the tumor, respectively.

**Supplementary Figure S3.** Agglomerative hierarchical clustering of samples based on the Bray-Curtis dissimilarity matrix calculated using rarefied ASV counts. Symbols on the leaves indicate patient identifiers, as shown in the legend. On-tumor samples are highlighted with a black border.

**Supplementary Figure S4.** Alpha diversity (ASV level Shannon diversity, top panel, and richness, bottom panel). Metrics were calculated based on singly rarefied ASV counts tables (subsampling depth of 25,000 total counts per sample). Data shown as boxplots and symbols reflect data from all patients, labeled as A-K. Alpha diversity in on- and off-tumor samples was not significantly different, as assessed using a linear mixed-effects model with the patient as a random effect and sample location (on- and off-tumor) as a fixed effect.

**Supplementary Figure S5.** Bubble plots of ASV wise differential abundances between on- and off-tumor samples. For this analysis, we calculated fold-differences in the abundance of ASVs between on- and off-tumor samples, considering all possible pairwise comparisons (that is, irrespective of the distance between the off-tumor samples and the tumors) and subsequently ranked ASVs according to the mean of the log_2_-transformed fold abundance differences. Symbol sizes are scaled according to ASV abundance (averaged across replicates) in the tumor samples and colors reflect phylum-level taxonomic assignments as indicated in the legend. Error bars indicate standard deviations of all possible pairwise comparisons. Facet labels indicate patient identifiers.

**Supplementary Figure S6.** Bar chart of the effect sizes of ASVs with significantly differential abundance (q-value of <0.1) between on- and off-tumor samples. Statistical analysis was performed using MaAsLin2 as described in the main Methods, using a linear mixed-effect model (patient as random effect and location, encoded as a categorical binary variable, as fixed effect). Q-values (that is, *P* values after correction for multiple testing) are indicated.

**Supplementary Figure S7.** Plots of the relative abundances for ASVs shown in the main figure for patient B. Dark-red symbols reflect on-tumor samples and off-tumor samples (shown in grey) are plotted according to their distance from the tumor.

**Supplementary Figure S7, cont’d**. Data are for patient G.

**Supplementary Figure S7, cont’d**. Data are for patient F.

**Supplementary Figure S8.** Novelty (as sequence identity to sequences in the LTP database, release LTP_01_2022) as a function of ASV abundance. For each ASV, the maximum abundance across all samples is plotted and colored according to the patient in which it was identified. ASVs with an abundance of ≥1% in at least one sample and with an identity of ≤97% to sequences in the LTP database are highlighted. The horizontal red line indicates 97% sequence identity.

**Supplementary Figure S8, cont’d.** Abundances of ASVs highlighted in the upper panel. Labels on the *y*-axis indicate ASV identifiers, taxonomic assignment against the Silva database, and best-matches against the LTP database, with identities in square brackets, separated by a vertical bar. Relative abundances across samples are shown for all patients, as indicated in the fill color legend.

**Supplementary Figure S9.** Abundance of all MAGs linked to ASVs by MarkerMAG. Symbols show abundances across all samples and are color-coded according to patient as indicated in the legend.

**
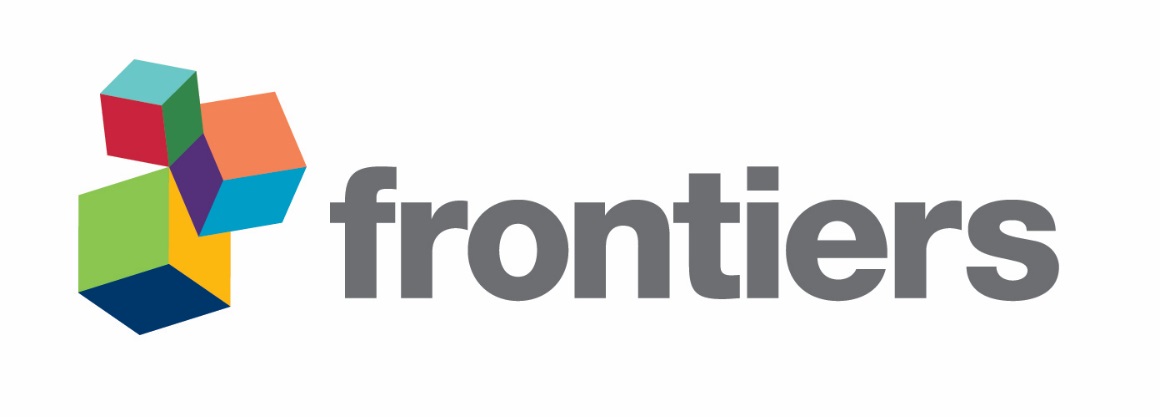
**
